# PKM1 is required for embryonic cardiomyocyte proliferation through energetic regulation of NFYa stability

**DOI:** 10.1093/nsr/nwaf408

**Published:** 2025-09-25

**Authors:** Dandan Zhang, Yansong Tang, Wen Ye, Danli Yang, Shengtang Qin, Juntao Liu, Nan Su, Rongrong Huang, Guangzheng Shi, Dachun Xu, Xiaochen Kou, Yanhong Zhao, Hong Wang, Shaorong Gao, Ke Wei, Lan Kang

**Affiliations:** Institute for Regenerative Medicine, Shanghai East Hospital, Shanghai Institute of Stem Cell Research and Clinical Translation, Shanghai Key Laboratory of Signaling and Disease Research, Frontier Science Center for Stem Cell Research, School of Life Sciences and Technology, Tongji University, Shanghai 200092, China; Institute for Regenerative Medicine, Shanghai East Hospital, Shanghai Institute of Stem Cell Research and Clinical Translation, Shanghai Key Laboratory of Signaling and Disease Research, Frontier Science Center for Stem Cell Research, School of Life Sciences and Technology, Tongji University, Shanghai 200092, China; Institute for Regenerative Medicine, Shanghai East Hospital, Shanghai Institute of Stem Cell Research and Clinical Translation, Shanghai Key Laboratory of Signaling and Disease Research, Frontier Science Center for Stem Cell Research, School of Life Sciences and Technology, Tongji University, Shanghai 200092, China; Institute of Cancer Stem Cell, Dalian Medical University, Dalian 116044, China; Institute of Cancer Stem Cell, Dalian Medical University, Dalian 116044, China; Institute for Regenerative Medicine, Shanghai East Hospital, Shanghai Institute of Stem Cell Research and Clinical Translation, Shanghai Key Laboratory of Signaling and Disease Research, Frontier Science Center for Stem Cell Research, School of Life Sciences and Technology, Tongji University, Shanghai 200092, China; Institute for Regenerative Medicine, Shanghai East Hospital, Shanghai Institute of Stem Cell Research and Clinical Translation, Shanghai Key Laboratory of Signaling and Disease Research, Frontier Science Center for Stem Cell Research, School of Life Sciences and Technology, Tongji University, Shanghai 200092, China; Department of Cardiology, Clinical Research Unit, Shanghai Tenth People’s Hospital, Tongji University School of Medicine, Shanghai 200092, China; Department of Cardiology, Clinical Research Unit, Shanghai Tenth People’s Hospital, Tongji University School of Medicine, Shanghai 200092, China; Department of Cardiology, Clinical Research Unit, Shanghai Tenth People’s Hospital, Tongji University School of Medicine, Shanghai 200092, China; Frontier Science Center for Stem Cell Research, Tongji University, Shanghai 200092, China; Frontier Science Center for Stem Cell Research, Tongji University, Shanghai 200092, China; Clinical and Translation Research Center of Shanghai First Maternity & Infant Hospital, School of Life Sciences and Technology, Tongji University, Shanghai 200092, China; Clinical and Translation Research Center of Shanghai First Maternity & Infant Hospital, School of Life Sciences and Technology, Tongji University, Shanghai 200092, China; Frontier Science Center for Stem Cell Research, Tongji University, Shanghai 200092, China; Institute for Regenerative Medicine, Shanghai East Hospital, Shanghai Institute of Stem Cell Research and Clinical Translation, Shanghai Key Laboratory of Signaling and Disease Research, Frontier Science Center for Stem Cell Research, School of Life Sciences and Technology, Tongji University, Shanghai 200092, China; Institute for Regenerative Medicine, Shanghai East Hospital, Shanghai Institute of Stem Cell Research and Clinical Translation, Shanghai Key Laboratory of Signaling and Disease Research, Frontier Science Center for Stem Cell Research, School of Life Sciences and Technology, Tongji University, Shanghai 200092, China; Frontier Science Center for Stem Cell Research, Tongji University, Shanghai 200092, China

**Keywords:** PKM1, cardiac development, cardiomyocyte proliferation, AMPK, NFYa

## Abstract

Pyruvate kinase M1 (PKM1) is a critical enzyme in glycolysis, particularly in high-energy-demand tissues like the heart. However, previous knockout strategies for PKM1 were confounded by compensatory upregulation of its low-activity splice variant, PKM2. Here, we generated a *Pkm1* mutant mouse model using a point mutation that eliminates PKM1 without compensatory PKM2 upregulation. Homozygous *Pkm1* mutants exhibited perinatal lethality associated with cardiac dysfunction, characterized by thin myocardium and reduced cardiomyocyte proliferation during mid-to-late gestation. We found that PKM1 sustains ATP levels to inhibit AMPK, which otherwise promotes NFYa phosphorylation and destabilization. NFYa, a transcription factor essential for cardiomyocyte proliferation, has been identified as a key mediator linking metabolic status to cell cycle. These findings identify the PKM1-AMPK-NFYa axis in energetic regulation of cardiomyocyte proliferation in the embryonic heart, offering new insights into the function of PKM1 and the broader impact of energy metabolism on cardiac development, while also shedding light on the potential metabolic underpinnings of congenital heart diseases.

## INTRODUCTION

In mammals, various tissues with drastically different energy demands employ different strategies to control energy supply and regulate cellular and organ response to changes in energy status [1–4]. In addition, diverse scenarios, such as developmental [5] and ageing progression [6], as well as physiological and pathological adaptations [7,8], add to the complexity of energy regulation of cellular behavior. Pyruvate kinases (PKs) are enzymes catalyzing the conversion of phosphoenolpyruvate (PEP) to pyruvate, which is a key step in glucose metabolism [9]. *Pkm* is a major gene encoding two different PKs, PKM1 and PKM2, which differ by one alternatively spliced exon [9]. PKM1 forms an active tetramer, enriched in tissues with high energy demands like muscle and brain, efficiently producing ATP [9,10]. Conversely, PKM2, expressed in cells with high synthetic needs such as embryonic and cancer cells, is less active and supports cell growth by diverting glycolytic intermediates to biosynthesis [9,11–13], as well as playing other non-metabolic roles [14,15].

The heart is the first functioning organ during mammalian development, and its energy-consuming contraction lasts throughout life [16]. During cardiac development, the heart’s growth requires significant metabolic gear-shifting events to accommodate [17] both the escalating energy demand for blood circulation through the fetus and the increasing need for biosynthesis to facilitate the hyperplastic and hypertrophic growth of cardiomyocytes [16]. Notably, from early to late gestation, energy metabolism in cardiomyocytes shifts from aerobic glycolysis to the TCA cycle to boost energy output [18], while after birth, fatty acid oxidation (FAO), an oxidative phosphorylation (OXPHOS) process using fatty acid as the major substrate, further augments the efficiency of energy production [19]. Thus, it is surprising that elimination of the specific splicing of *Pkm1*, which is responsible for efficient pyruvate production from glucose, shows no cardiac phenotype [20], while cardiac-specific knockout (KO) of *Pkm1* also displayed no obvious cardiac defect without pathological challenge [21].

The balance between PKM1 and PKM2 is crucial for regulating cellular energy production and synthetic activities, and is under complex regulation of alternative splicing [11,12,22–24]. Employing conditional knockout of *Pkm1* or *Pkm2* targeting the isoform-specific exons, previous reports emphasized the role of PKM1 and PKM2 in metabolic remodeling in tumor cells [20,21,25–27], but the survivability of *Pkm1* or *Pkm2* KO mice may be mainly due to elevated expression of the other isoform.

In our research, we provided new phenotypes of PKM1 ablation, in the absence of PKM2 compensation. Through comprehensive phenotypic and mechanistic investigations, we unveiled PKM1’s crucial role in facilitating cardiomyocyte proliferation during late gestation. Furthermore, we identified NFYa as a pivotal transcription factor that links PKM1 and energy metabolism to the transcriptional regulation of genes involved in cell proliferation. This study offers novel insights into the metabolic control of cardiomyocyte proliferation and its implications for cardiac development.

## RESULTS

### *Pkm1* deletion causes neonatal lethality with cardiac dysfunction

**Figure 1.**
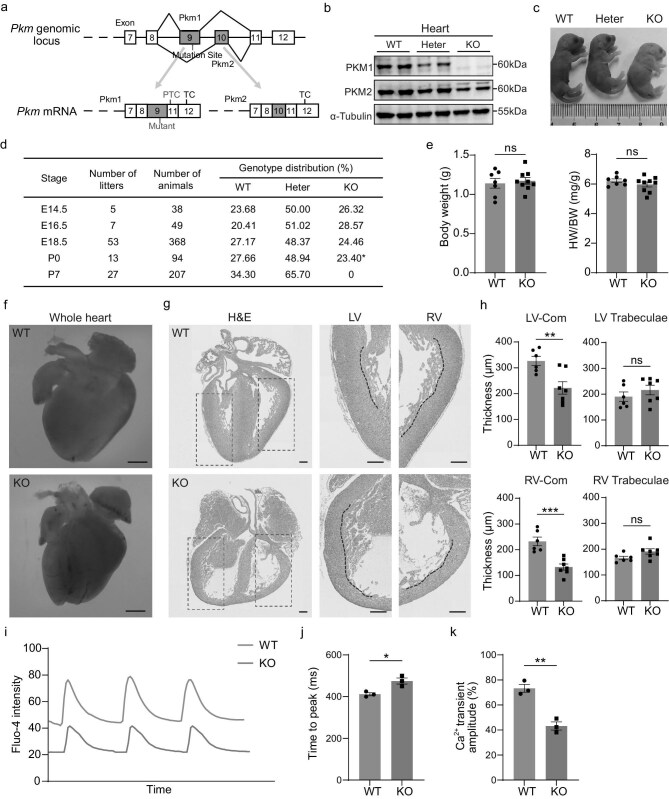
*Pkm1* deletion causes neonatal lethality with cardiac dysfunction. (a) Schematic of *Pkm1* knockout (KO) mouse model. PTC, premature termination codon. TC, termination codon. (b) Western blot analysis of PKM1, PKM2 protein levels in hearts from wild-type (WT), heterozygous (Heter) and KO embryos at embryonic days (E) 18.5. (c) Representative photograph of pups at postnatal day 0 (P0) obtained from heterozygote intercrosses. (d) Ratio of observed mice genotype at embryonic stage (E14.5, *N* = 5 female mice, *n *= 38 embryos; E16.5, *N* = 7 female mice, *n *= 49 embryos; E18.5, *N* = 53 female mice, *n *= 368 embryos) or after birth (P0, *N* = 13 litters, *n *= 94 pups; P7, *N* = 27 litters, *n *= 207 pups) following heterozygote intercrosses. Asterisk indicates that newborn homozygous knockout pups die within hours of birth. (e) Body weight and ratios of heart weight to body weight (HW/BW) in E18.5 WT and KO embryos (*n *= 7 embryos for WT, 9 embryos for KO). (f) Representative images of the whole heart at E18.5. Scale bars, 500 μm. (g) Hematoxylin-eosin (H&E) staining of WT and KO hearts at E18.5. LV, left ventricle. RV, right ventricle. Scale bars, 200 μm. (h) Quantification of left ventricular compact myocardium (LV-Com), LV trabeculae, right ventricular compact myocardium (RV-Com) and RV trabeculae thicknesses of E18.5 WT and KO hearts (*n* = 6 hearts for WT, 7 hearts for KO). (i) Representative calcium transient traces from cardiomyocytes isolated from E18.5 WT and KO hearts. (j) Quantification of time to peak calcium fluorescence (*n* = 3 wells from 6 hearts per group). (k) Quantification of Ca^2+^ transient amplitude (*n* = 3 wells from 6 hearts per group). Two-tailed unpaired Student’s t test was performed (e, h, j, k). All quantitative data are expressed as mean ± SEM. *P < 0.05, **P < 0.01, ***P < 0.001, ns: not significant.

To investigate the role of PKM1 in development, we generated a *Pkm1-*KO mouse using CRISPR/Cas9 technology targeting the *Pkm1*-specific exon. A 26 bp deletion in exon 9 of *Pkm* resulted in the formation of a premature termination codon (PTC) in the *Pkm1* transcript (Fig. 1a). PCR and western blot confirmed the absence of PKM1 without compensatory upregulation of PKM2 in hearts and other tissues examined of perinatal *Pkm1* homozygous deletion (*Pkm1*^−/−^) mice (Fig. 1b and Fig. S1a, b). *Pkm1* heterozygous deletion mice (*Pkm1*^+/−^) were able to survive to adulthood and be fertile, without obvious defects. However, no *Pkm1*^−/−^ mice survived to adulthood among the offspring of *Pkm1*^+/−^ parents. Further investigation into the development of these mice revealed that *Pkm1*^−/−^ embryos followed the expected Mendelian ratio from embryonic days (E) 14.5 to E18.5, whereas neonatal *Pkm1*^−/−^ (hereafter referred as KO) mice died within a few hours of birth (Fig. 1c, d). To rule out potential off-target effects of the CRISPR/Cas9 strategy, the top 15 predicted off-target loci were examined by both Sanger sequencing and TIDE analysis, with no detectable mutations found in KO embryos (Table S2).

Neonatal death is often a sign of cardiopulmonary dysfunction. Histological analysis revealed no morphological abnormalities or difference in thickness in the diaphragms of KO embryos at E18.5, compared to littermate wildtype (WT) embryos (Fig. S1c, d). E18.5 KO embryos exhibited no abnormalities in lung morphology and no alterations in the expression levels of marker genes associated with pulmonary lesions (Fig. S1e, f). Additionally, a floating test showed that the lungs of KO neonates were inflated, suggesting a normal pulmonary function after birth (Fig. S1g).

Given that KO neonates died immediately after birth with an intact pulmonary function, we focused on the hearts of E18.5 KO embryos immediately before birth. There were no significant differences in body weight or heart weight to body weight ratio between WT and KO embryos at E18.5 (Fig. 1e), however, histological analysis revealed that the ventricular compact myocardium in KO hearts was significantly thinner compared to WT hearts (Fig. 1f–h), while the thickness of the trabeculae showed no significant difference between the two groups (Fig. 1h). The thickness of the ventricular wall is critical for normal cardiac function, and thinning of the ventricular wall typically leads to impaired contractile function, reduced cardiac output and compromised circulation. In addition, we observed atrial septal defects (ASDs) in E18.5 KO embryos, a form of congenital heart disease (CHD) that can further disrupt intracardiac blood flow and systemic circulation (Fig. S1h).

To further evaluate whether these structural defects resulted in functional impairment of the myocardium, we isolated cardiomyocytes from E18.5 WT and KO hearts and performed calcium transient analysis using Fluo-4 acetoxymethyl ester (Fluo-4 AM) staining. Compared with WT cardiomyocytes, *Pkm1*-KO cardiomyocytes exhibited a markedly prolonged time to peak and reduced calcium transient amplitude, indicating impaired excitation-contraction coupling and weakened contractile capacity (Fig. 1i–k).

In brief, *Pkm1* deletion, without compensatory PKM2 upregulation, results in neonatal lethality in mice and leads to both significant structural and functional abnormalities in the heart.

### PKM1 is required for proliferation of embryonic cardiomyocytes

**Figure 2.**
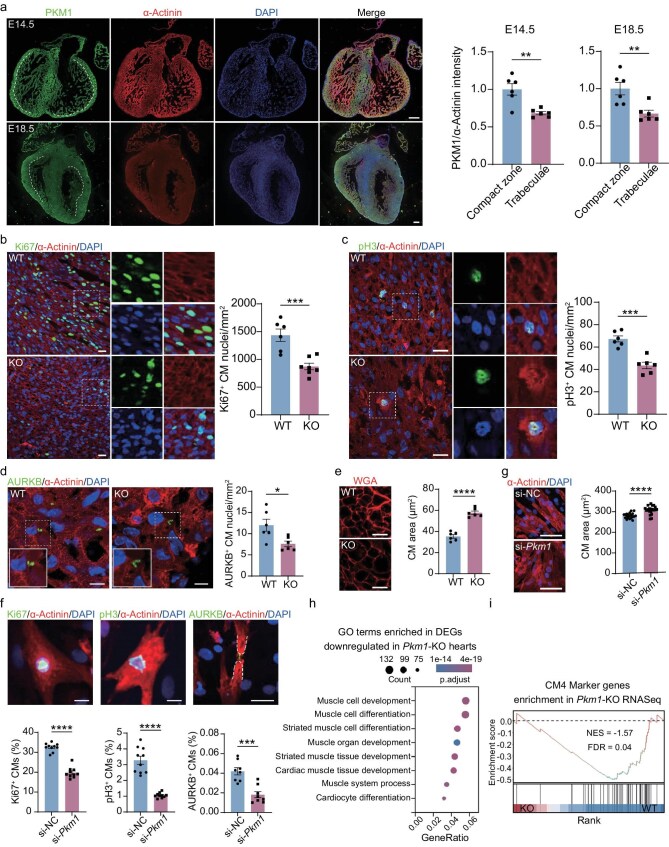
PKM1 is required for proliferation of embryonic cardiomyocytes. (a) Representative images of PKM1 staining and quantification of PKM1 protein levels in compact zone and trabeculae zone of E14.5 and E18.5 hearts (*n* = 6 hearts per group). Scale bars, 200 μm. (b) Representative images of Ki67 staining and quantification of Ki67^+^ nuclei per mm^2^ area of E18.5 WT and KO hearts (*n* = 6 hearts for WT, 7 hearts for KO). Scale bars, 20 μm. (c) Representative images of pH3 staining and quantification of pH3^+^ nuclei per mm^2^ area of E18.5 WT and KO hearts (*n* = 6 hearts per group). Scale bars, 20 μm. (d) Representative images of AURKB staining and quantification of AURKB^+^ nuclei per mm^2^ area of E18.5 WT and KO hearts (*n* = 6 hearts per group). Scale bars, 10 μm. (e) Representative images of wheat germ agglutinin (WGA) staining and quantification of mean cardiomyocyte cross-section area (CSA) of E18.5 WT and KO hearts (*n* = 6 hearts per group). Scale bars, 10 μm. (f) Representative images of Ki67, pH3, AURKB staining and quantification of Ki67^+^ (*n* = 10 wells per group), pH3^+^ (*n* = 10 wells per group), AURKB^+^ (*n* = 8 wells per group) cardiomyocytes (CMs) over total CMs in neonatal rat ventricular cardiomyocytes (NRVCs) 48 h after being transfected with si-negative control (NC) or si-*Pkm1*. Scale bars, 50 μm. (g) Representative images of α-Actinin staining and quantification of mean area in NRVCs 48 h after being transfected with si-NC or si-*Pkm1* (*n* = 20 wells per group). Scale bars, 100 μm. (h) Gene ontology (GO) analysis of the differentially expressed genes (DEGs) that were downregulated in KO hearts compared to WT hearts at E18.5 (*n* = 3 biological replicates per group). (i) Gene set enrichment analysis (GSEA) plot showing the upregulation of CM4 marker genes in WT hearts. NES, normalized enrichment score. FDR, false discovery rate. Two-tailed unpaired Student’s t test was performed (a–g). All quantitative data are expressed as mean ± SEM. *P < 0.05, **P < 0.01, ***P < 0.001, ****P < 0.0001, ns: not significant.

In line with the observed thinning of the compact ventricular wall in KO embryos, we found that from mid-gestation to the perinatal period, PKM1 expression in the compact myocardium was higher than in the trabecular region (Fig. 2a and Fig. S2a), and the level of PKM1 in cardiomyocytes increased in late gestation while PKM2 remained constant (Fig. S2b). To investigate whether the thinning of the compact myocardium in KO embryos was due to reduced cell proliferation or increased cell death, we examined the level of several proliferation and apoptosis markers in cardiomyocytes of both WT and KO embryos. We observed a significant reduction in the number of Ki67- and pH3-positive cardiomyocytes, and AURKB-positive cleavage furrows between two separating cardiomyocytes, per unit area in the compact myocardium of KO hearts (Fig. 2b–d). On the other hand, terminal deoxynucleotidyl transferase dUTP nick end labeling (TUNEL) staining showed no significant difference in cardiomyocyte death between the two groups (Fig. S3a). Moreover, wheat germ agglutinin (WGA) staining showed an increase in cardiomyocyte size in KO embryos (Fig. 2e), suggesting that compensatory hypertrophic growth of cardiomyocytes contributed to the sustained heart weight in KO mice (Fig. 1e).

Next, we sought to clarify when the reduction in cardiomyocyte proliferation began in KO embryos. Hearts at E12.5, E14.5 and E16.5 were collected and stained for Ki67 to measure cardiomyocyte proliferation. The results showed no differences in cardiomyocyte proliferation in E12.5 and E14.5 KO embryos compared to WT controls, and the decrease in proliferation occurred in late gestation (∼E16.5) (Fig. S3b–d), which correlated with the changes in PKM1 expression we observed at different stages of cardiac development (Fig. 2a and Fig. S2a, b).

To confirm our *in vivo* findings, we knocked down *Pkm1* in neonatal rat ventricular cardiomyocytes (NRVCs) using siRNA [21] (Fig. S4a, b). Compared to si-negative control (NC), si-*Pkm1* led to reduced cardiomyocyte proliferation (Fig. 2f) and increased cell size (Fig. 2g), consistent with the phenotypes observed in KO hearts (Fig. 2b–e).

To further investigate the molecular mechanisms underlying the effects of PKM1 ablation, we performed RNA-seq analysis on ventricular tissue at E18.5 to evaluate the transcriptomic differences between WT and KO hearts (Fig. S4c, d). Differentially expressed genes (DEGs) downregulated in KO hearts were enriched in muscle cell development- and differentiation-associated processes (Fig. 2h), suggesting impaired cardiac development. A previous study using single-nucleus RNA-seq (snRNA-seq) of postnatal day 1 to day 8 mouse hearts identified five cardiomyocyte clusters (CM1-CM5), among which the cluster CM4 represents a cardiomyocyte population with proliferative potential [28] (Fig. S4e, f). Gene set enrichment analysis (GSEA) showed an overall downregulation of CM4 marker genes in *Pkm1*-KO hearts (Fig. 2i), which coincided with our results of reduced cardiomyocyte proliferation upon *Pkm1* knockout and knockdown (Fig. 2b–g).

Taken together, these data demonstrate that elimination of PKM1 results in reduced cardiomyocyte proliferation and disrupted normal cardiac development.

### *Pkm1* deletion causes metabolic dysregulation and impairs mitochondrial respiration

**Figure 3.**
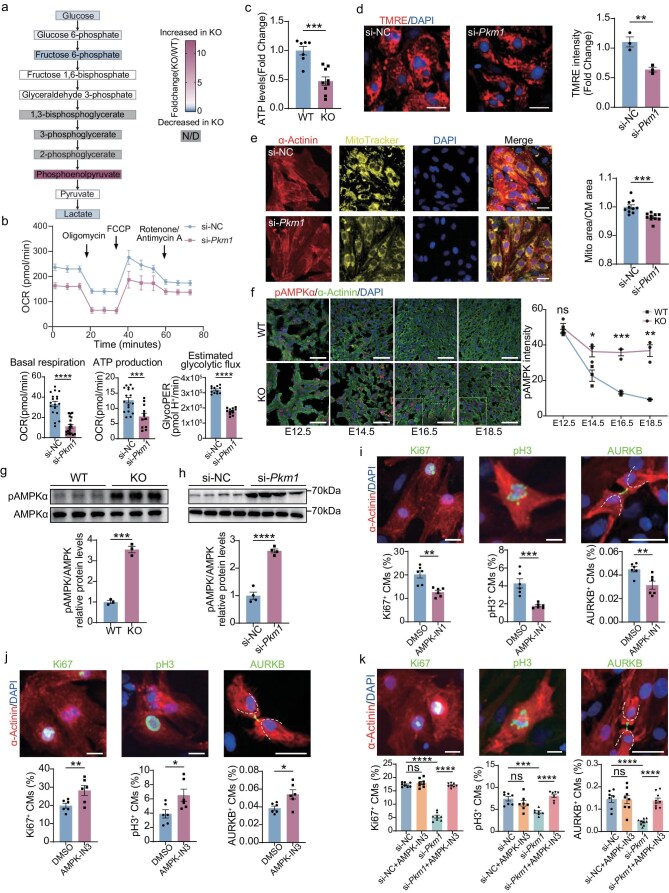
*Pkm1* deletion causes metabolic dysregulation and impairs mitochondrial respiration. (a) Foldchange of glycolytic metabolites in E18.5 WT and KO hearts. Box color depicts the fold-change (KO/WT). Gray-marked metabolites were not detected (N/D). (b) Analysis and quantification of mitochondrial oxygen consumption rates (OCRs) in NRVCs 48 h after being transfected with si-NC or si-*Pkm1* (*n* = 19 wells/si-NC, 18 wells/si-*Pkm1*). FCCP, carbonyl cyanide-p-trifluoromethoxyphenylhydrazone. Drug addition is indicated. (c) ATP levels of E18.5 WT and KO hearts (*n* = 7 hearts for WT, 9 hearts for KO). Data are normalized to total proteins. (d) Representative images of tetramethylrhodamine ethyl ester (TMRE) staining and quantification of mitochondrial membrane potential in NRVCs 48 h after being transfected with si-NC or si-*Pkm1* (*n* = 3 wells per group). Scale bars, 50 μm. (e) Representative images of MitoTracker staining and quantification of mitochondrial distribution in NRVCs 48 h after being transfected with si-NC or si-*Pkm1* (*n* = 10 wells per group). Scale bars, 50 μm. (f) Representative images and quantification of phosphorylated AMPKα^T172^ (pAMPK) in WT and KO hearts at E12.5, E14.5, E16.5, and E18.5 (*n* = 3 hearts per group). Scale bars, 50 μm. (g) Western blot analysis and quantification of pAMPK, AMPK protein levels in E18.5 WT and KO hearts (*n* = 3 hearts per group). (h) Western blot analysis and quantification of pAMPK, AMPK protein levels in NRVCs 48 h after being transfected with si-NC or si-*Pkm1* (*n* = 4 wells per group). (i) Representative images of Ki67, pH3, AURKB staining and quantification of Ki67^+^ (*n* = 6 wells per group), pH3^+^ (*n* = 6 wells per group), AURKB^+^ (*n* = 6 wells per group) CMs over total CMs in NRVCs treated with dimethyl sulfoxide (DMSO) or AMPK-IN1 (2 μM) for 6 h. Scale bars, 50 μm. (j) Representative images of Ki67, pH3, AURKB staining and quantification of Ki67^+^ (*n* = 6 wells per group), pH3^+^ (*n* = 6 wells per group), AURKB^+^ (*n* = 6 wells per group) CMs over total CMs in NRVCs treated with DMSO or AMPK-IN3 (5 μM) for 24 h. Scale bars, 50 μm. (k) Representative images of Ki67, pH3, AURKB staining and quantification of Ki67^+^ (*n* = 8 wells per group), pH3^+^ (*n* = 7 wells per group), AURKB^+^ (*n* = 7 wells/si-NC, 8 wells/si-NC + AMPK-IN3, 9 wells/si-*Pkm1*, 9 wells/si-*Pkm1 *+ AMPK-IN3) CMs over total CMs in NRVCs transfected with si-NC or si-*Pkm1*, treated with DMSO or AMPK-IN3 (2 μM) for 24 h. Scale bars, 50 μm. Two-tailed unpaired Student’s t test was performed (b–j). One-way ANOVA, Tukey’s Multiple Comparison Test was performed (k). All quantitative data are expressed as mean ± SEM. *P < 0.05, **P < 0.01, ***P < 0.001, ****P < 0.0001, ns: not significant.

Since PKM1 is a key enzyme in the glycolysis pathway, we sought to clarify the regulatory role of PKM1 in cardiomyocyte metabolism. Untargeted metabolomic analysis was performed on hearts from E18.5 KO and WT embryos, and Kyoto encyclopedia of genes and genomes (KEGG) analysis of the differentially abundant metabolites showed that the loss of PKM1 affected the metabolism of amino acids, nucleotides, lipids and carbon, which is consistent with a systemically defective metabolism (Fig. 3a and Fig. S5a). More importantly, as the direct substrate of PKM1, PEP was markedly accumulated in the PKM1-deficient hearts, suggesting impaired glycolysis (Fig. 3a). Consistently, Seahorse XF analysis revealed a significant reduction in glycolytic proton efflux rate (GlycoPER) in si-*Pkm1*-treated NRVCs, together with downregulated basal oxygen consumption rates (OCRs) and ATP production (Fig. 3b).

Pyruvate, mainly generated by PKM1, serves as the principal fuel for mitochondrial energy production in embryonic hearts. In *Pkm1*-KO hearts, ATP levels were significantly reduced (Fig. 3c), and si-*Pkm1*-treated NRVCs exhibited a marked decline in mitochondrial membrane potential, as measured by tetramethylrhodamine ethyl ester (TMRE), indicating mitochondrial depolarization (Fig. 3d). Additionally, MitoTracker and α-Actinin staining revealed that content of mitochondria in cardiomyocytes, measured as the ratio of mitochondrial area to cardiomyocyte area, was significantly reduced in si-*Pkm1*-treated NRVCs (Fig. 3e).

As elimination of PKM1 led to a significant reduction of ATP production in both embryonic mouse hearts and NRVCs, we examined the activity of AMP-activated protein kinase (AMPK), a key cellular energy sensor [29]. Upon energy starvation, the α subunit of AMPK is phosphorylated (pAMPKα) at Thr172 and the AMPK complex is activated [30]. We used immunofluorescence staining of pAMPKα (Thr172) to delineate the dynamics of AMPK activation during cardiac development. In WT hearts, the pAMPK signal was highest at E12.5, sharply declined by E14.5, and was almost diminished by E18.5 (Fig. 3f), consistent with reported developmental AMPK inactivation in the embryonic heart [18]. Notably, *Pkm1*-KO hearts exhibited a distinct AMPK activation trajectory: while the pAMPK signal was comparable to WT at E12.5, the reduction at E14.5 was moderate and it remained detectable through E18.5 (Fig. 3f). The elevation of pAMPK was pronounced from E16.5 onward in *Pkm1*-KO hearts, which coincided with the onset of cardiomyocyte proliferation defects in KO hearts starting at late gestation (∼E16.5) (Fig. S3b–d). Western blot analysis confirmed significantly increased AMPKα Thr172 phosphorylation in both KO hearts and NRVCs with *Pkm1* knockdown (Fig. 3g, h), indicating AMPK activation.

To explore whether AMPK activity is involved in regulating cardiomyocyte proliferation, we treated NRVCs with AMPK activator AMPK-IN1 and inhibitor AMPK-IN3, respectively. Compared to the control group, AMPK activation led to decreased NRVC proliferation (Fig. 3i), while AMPK inhibition enhanced NRVC proliferation (Fig. 3j and Fig. S6a), suggesting AMPK activity negatively regulates cardiomyocyte proliferation.

To determine whether PKM1 regulates cardiomyocyte proliferation through AMPK activity, we treated these cardiomyocytes with AMPK inhibitor AMPK-IN3 after knockdown of *Pkm1* in NRVCs. Percentages of Ki67- and pH3-positive cardiomyocytes, and AURKB-positive cleavage furrows between two separating cardiomyocytes were reduced in *Pkm1* knockdown cardiomyocytes, but were all restored by AMPK-IN3 at a concentration that does not affect baseline proliferation levels (Fig. 3k and Fig. S6a), demonstrating that AMPK inhibition rescued cardiomyocyte proliferation defects induced by PKM1 loss. These results suggest that AMPK activation mediates the effect of PKM1 loss-of-function on suppressing cardiomyocyte proliferation.

### NFYa is a critical regulator of cardiomyocyte proliferation downregulated upon *Pkm1* deletion

**Figure 4.**
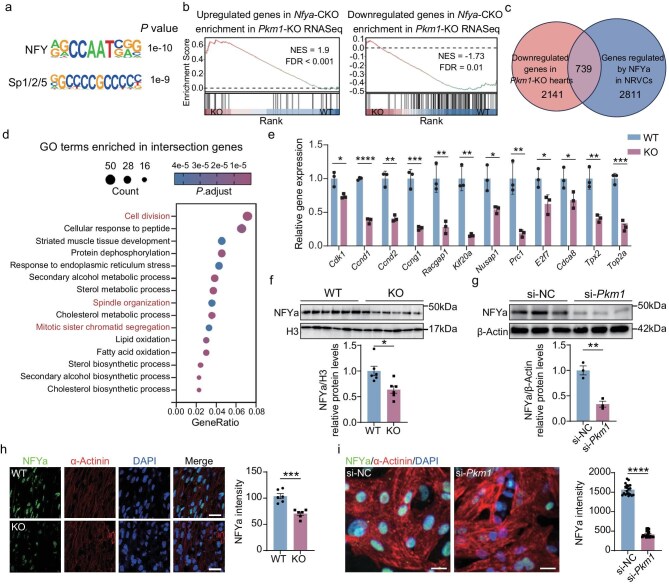
NFYa is a critical regulator of cardiomyocyte proliferation downregulated upon *Pkm1* deletion. (a) Motif analysis of downregulated genes in *Pkm1*-KO hearts at E18.5. (b) GSEA plot of RNA-seq data from *Nfya* conditional knockout (CKO) hearts, comparing the results to those of *Pkm1*-KO RNA-seq data. (c) Venn plot showing the overlap of downregulated genes in *Pkm1*-KO hearts and genes regulated by NFYa in NRVCs. (d) GO analysis of the overlapping genes from (c). (e) qRT-PCR analysis of NFYa target genes enriched in the ‘cell division’ GO term in E18.5 WT and KO hearts (*n* = 3 hearts per group). (f) Western blot analysis and quantification of NFYa protein levels in E18.5 WT and KO hearts (*n* = 6 hearts per group). (g) Western blot analysis and quantification of NFYa protein levels in NRVCs 48 h after being transfected with si-NC or si-*Pkm1* (*n* = 3 wells per group). (h) Representative images of NFYa staining and quantification of NFYa protein levels in E18.5 WT and KO hearts (*n* = 6 hearts per group). Scale bars, 20 μm. (i) Representative images of NFYa staining and quantification of NFYa protein levels in NRVCs 48 h after being transfected with si-NC or si-*Pkm1* (*n* = 20 wells per group). Scale bars, 50 μm. Two-tailed unpaired Student’s t test was performed (e–i). All quantitative data are expressed as mean ± SEM. *P < 0.05, **P < 0.01, ***P < 0.001, ****P < 0.0001, ns: not significant.

AMPK is a kinase with diverse substrates and downstream effectors, and transcription factors must be involved to alter transcription programs of proliferation genes upon energy shortage and AMPK activation. To uncover key transcription factors regulating genes downregulated in KO mouse hearts, we performed motif enrichment analysis using hypergeometric optimization of motif enrichment (HOMER) on the promoter regions of these genes. NFY and Sp family transcription factors were suggested to be associated with the regulation of these genes (Fig. 4a). NFYa has been considered as a key transcription factor regulating embryonic and perinatal cardiomyocyte proliferation [28,31], and its interaction with Sp transcription factors has also been reported [31], thus making NFYa a particularly compelling candidate for the downstream transcriptional effector of PKM1.

To dissect the relationship between PKM1 and NFYa, we conducted GSEA of up- and down-regulated genes in cardiomyocyte-specific *Nfya* knockout (*Nfya*-CKO) hearts [31] in *Pkm1-*KO RNA-seq data. Interestingly, genes upregulated in *Nfya*-CKO hearts were significantly upregulated in *Pkm1*-KO hearts (NES = 1.9, FDR <0.01), while genes downregulated in *Nfya*-CKO hearts were also significantly downregulated in *Pkm1*-KO hearts (NES = −1.73, FDR <0.01) (Fig. 4b). This strong correlation suggests that NFYa may play a central role in modulating the transcriptional changes upon *Pkm1* deletion. In addition, we analyzed the overlapping genes downregulated in *Pkm1*-KO hearts and occupied by NFYa in cardiomyocytes [31] (Fig. 4c). Gene ontology (GO) enrichment analysis of these 739 intersecting genes revealed that they were significantly enriched in cell cycle-related pathways, such as cell division, spindle organization and mitotic sister chromatid segregation (Fig. 4d, e), suggesting that NFYa may be the downstream transcription factor for PKM1 to affect cardiomyocyte proliferation.

To clarify the regulatory role of PKM1 on NFYa, we assessed *Nfya* mRNA and NFYa protein levels following *Pkm1* knockout and knockdown. There was no alteration in the transcriptional level of *Nfya* in KO hearts or in NRVCs treated with si-*Pkm1* (Fig. S7a, b). However, western blot showed a significant decrease in NFYa protein level in KO embryonic hearts compared to WT controls (Fig. 4f). A similar reduction in NFYa protein was observed in NRVCs with *Pkm1* knockdown (Fig. 4g). Immunofluorescence staining coincided with these findings, showing decreased NFYa protein levels in both KO embryonic cardiomyocytes and *Pkm1*-knockdown NRVCs relative to their respective controls (Fig. 4h, i). In addition, we also observed higher expression of NFYa in the compact myocardium compared to the trabecular region (Fig. S7c), which corresponds to the pattern of PKM1 (Fig. 2a). Collectively, these results indicate that PKM1 regulates the protein levels of NFYa in cardiomyocytes, rather than regulating its transcription.

### AMPK activation led to NFYa degradation upon *Pkm1* deletion

As we found that ablation of PKM1 led to the activation of AMPK and decreased abundance of NFYa protein, we sought to figure out the relationship between AMPK activity and NFYa level.

**Figure 5.**
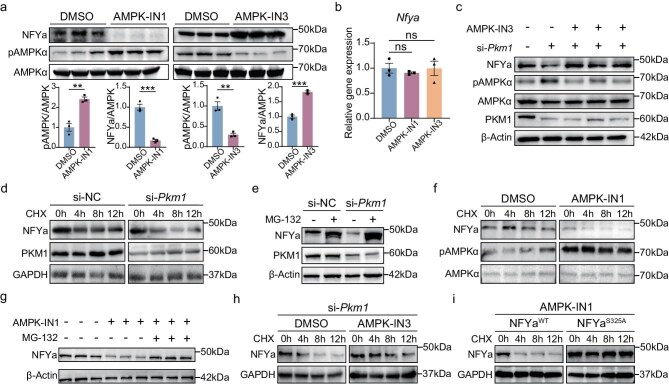
AMPK activation led to NFYa degradation upon *Pkm1* deletion. (a) Western blot analysis and quantification of NFYa, pAMPK protein levels in NRVCs treated with DMSO, AMPK-IN1(2 μM, for 6 h) or AMPK-IN3 (5 μM, for 24 h) (*n* = 3 wells per group). (b) qRT-PCR analysis of *Nfya* mRNA levels in NRVCs treated with DMSO, AMPK-IN1(2 μM, for 6 h) or AMPK-IN3 (5 μM, for 24 h) (*n* = 3 wells per group). (c) Western blot analysis of NFYa, pAMPK, AMPK protein levels in NRVCs transfected with si-NC or si-*Pkm1*, treated with DMSO or AMPK-IN3 (5 μM) for 24 h. (d) Western blot analysis of NFYa protein levels in NRVCs transfected with si-NC or si-*Pkm1*, treated with cycloheximide (CHX, 1 mM). (e) Western blot analysis of NFYa protein levels in NRVCs transfected with si-NC or si-*Pkm1*, treated with DMSO or MG-132 (10 μM) for 24 h. (f) Western blot analysis of NFYa, pAMPK protein levels in NRVCs treated with DMSO or AMPK-IN1 (2 μM) in the presence of CHX (1 mM). (g) Western blot analysis of NFYa protein levels in NRVCs treated with DMSO, AMPK-IN1 (2 μM, for 6 h) or MG-132 (10 μM, for 12 h) (*n* = 3 wells per group). (h) Western blot analysis of NFYa protein levels in NRVCs transfected with si-*Pkm1*, treated with DMSO or AMPK-IN3 (5 μM) in the presence of 1 mM CHX. (i) Western blot analysis of NFYa protein levels in WT and mutant NRVCs treated with CHX (1 mM). Two-tailed unpaired Student’s t test was performed (a). One-way ANOVA, Tukey’s Multiple Comparison Test was performed (b). All quantitative data are expressed as mean ± SEM. *P < 0.05, **P < 0.01, ***P < 0.001, ****P < 0.0001, ns: not significant.

First, we tested whether AMPK activity affects NFYa protein level. Treatment with the AMPK activator AMPK-IN1 in NRVCs led to a significant reduction in NFYa levels, while AMPK inhibitor AMPK-IN3 was the opposite (Fig. 5a). At the same time, the mRNA level of *Nfya* remained unchanged (Fig. 5b), suggesting that AMPK affected NFYa protein level rather than its transcription. To examine whether AMPK activation mediates the effect of PKM1 loss-of-function on NFYa protein, we treated NRVCs with si-*Pkm1* together with AMPK-IN3. *Pkm1* knockdown led to increased AMPK phosphorylation and reduced NFYa protein, while these effects were eliminated by AMPK-IN3 (Fig. 5c). This result supports the idea that PKM1 deficiency activates AMPK, which in turn regulates NFYa protein level.

To further explore the mechanism by which AMPK regulates NFYa abundance, we first examined how NFYa is degraded. In the presence of cycloheximide (CHX) which inhibits protein synthesis, treatment with the proteasome inhibitor MG-132, but not lysosome inhibitor E64d, blocked NFYa degradation (Fig. S8a), indicating that NFYa is degraded via the proteasome pathway but not the lysosomal pathway. Then we examined the level of NFYa protein in NRVCs in the presence of CHX (Fig. 5d) or MG-132 (Fig. 5e) with or without *Pkm1* knockdown, and found that NFYa was degraded more rapidly in PKM1-deficient NRVCs when protein synthesis was inhibited (Fig. 5d), while blocking proteasome with MG-132 rescued NFYa protein levels in both si-NC- and si-*Pkm1* treated cells to a comparable extent (Fig. 5e), indicating that PKM1 deficiency does not affect NFYa protein synthesis but rather its post-translational degradation through the proteasome pathway.

To clarify the role of AMPK in NFYa degradation, we activated AMPK using AMPK-IN1 in NRVCs, and observed accelerated NFYa degradation (Fig. 5f), an effect that was blunted by MG-132 (Fig. 5g), indicating that AMPK activation enhances proteasomal degradation of NFYa. Conversely, inhibiting AMPK via AMPK-IN3 slowed NFYa degradation in PKM1-deficient NRVCs (Fig. 5h), further supporting the notion that PKM1 stabilizes NFYa by inhibiting AMPK.

Finally, as AMPK is a kinase involved in several signaling pathways, we investigated whether posttranslational modification, such as phosphorylation, is responsible for NFYa degradation promoted by AMPK. Utilizing PhosphoSitePlus [32], we identified serine 325 as the primary candidate for phosphorylation within NFYa. Thus, we generated a phosphorylation-resistant S325A NFYa mutant and assessed its stability in response to AMPK activation. The S325A NFYa mutant exhibited markedly enhanced stability compared to the wildtype NFYa (Fig. 5i), indicating that phosphorylation at S325, potentially mediated by AMPK, is essential for the proteasomal degradation of NFYa.

Together, these results revealed that AMPK plays a key role in regulating NFYa stability through proteasomal degradation, with its activity modulated by PKM1 abundance. Depletion of PKM1 triggers AMPK activation, which in turn promotes NFYa degradation through S325 phosphorylation. Conversely, AMPK inhibition not only preserves NFYa levels but also restores cardiomyocyte proliferation impaired by PKM1 deficiency. These observations underscore the critical role of AMPK-dependent regulation of NFYa stability in cardiomyocytes.

### NFYa overexpression rescues proliferation and structural defects in *Pkm1* knockout hearts

**Figure 6.**
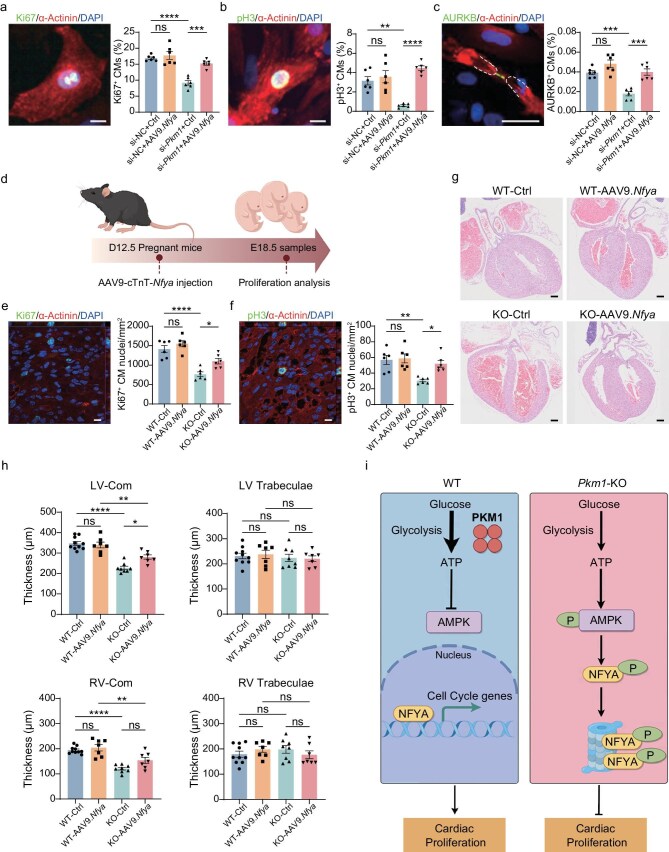
NFYa overexpression rescues proliferation and structural defects in *Pkm1* knockout hearts. (a) Representative image of Ki67 staining and quantification of Ki67^+^ CMs over total CMs in NRVCs transfected with si-NC or si-*Pkm1*, with or without overexpression of NFYa for 48 h (*n* = 6 wells per group). Scale bar, 50 μm. (b) Representative image of pH3 staining and quantification of pH3^+^ CMs over total CMs in NRVCs transfected with si-NC or si-*Pkm1*, with or without overexpression of NFYa for 48 h (*n* = 6 wells per group). Scale bar, 50 μm. (c) Representative image of AURKB staining and quantification of AURKB^+^ CMs over total CMs in NRVCs transfected with si-NC or si-*Pkm1*, with or without overexpression of NFYa for 48 h (*n* = 6 wells per group). Scale bar, 50 μm. (d) Schematic diagram of CM proliferation analysis post AAV9-cTnT-*Nfya* injection. AAV9-cTnT-*Nfya* was administered via tail vein injection to pregnant mice at E12.5, and embryonic hearts were harvested at E18.5 for analysis. (e) Representative image of Ki67 staining and quantification of Ki67^+^ nuclei per mm^2^ area of E18.5 WT and KO hearts with or without overexpression of NFYa (*n* = 6 hearts per group). AAV9 was administered at E12.5. Scale bar, 10 μm. (f) Representative image of pH3 staining and quantification of pH3^+^ nuclei per mm^2^ area of E18.5 WT and KO hearts with or without overexpression of NFYa (*n* = 6 hearts per group). AAV9 was administered at E12.5. Scale bar, 10 μm. (g) H&E staining of WT and KO hearts with or without overexpression of NFYa at E18.5. AAV9 was administered at E12.5. Scale bars, 200 μm. (h) Quantification of left ventricular compact myocardium (LV-Com), LV trabeculae, right ventricular compact myocardium (RV-Com) and RV trabeculae thicknesses of hearts from (g) (*n* = 10 hearts for WT-Ctrl, 7 hearts for WT-AAV9.*Nfya*, 8 hearts for KO-Ctrl, 7 hearts for KO-AAV9.*Nfya*). (i) Model of PKM1 regulating CM proliferation by inhibiting AMPK’s degradation of transcription factor NFYa. One-way ANOVA, Tukey’s Multiple Comparison Test was performed (a–c, e–f, h). All quantitative data are expressed as mean ± SEM. *P < 0.05, **P < 0.01, ***P < 0.001, ****P < 0.0001, ns: not significant.

To functionally test whether NFYa is responsible for sustaining cardiomyocyte proliferation downstream of PKM1, we overexpressed NFYa via AAV9 (Fig. S9a) in *Pkm1*-knockdown NRVCs, and assessed cardiomyocyte proliferation. NFYa overexpression was able to restore the proportion of Ki67- and pH3-positive cardiomyocytes, and AURKB-positive cleavage furrows between dividing cardiomyocytes, which were all decreased by si-*Pkm1* (Fig. 6a–c), showing that NFYa overexpression can rescue the proliferation defects caused by *Pkm1* knockdown, thus supporting that NFYa acts downstream of PKM1 to sustain cardiomyocyte proliferation.

Furthermore, we aimed to determine whether NFYa overexpression could also rescue the proliferative and structural defects in *Pkm1*-KO hearts. At E12.5, we administered AAV9-cTnT-*Nfya* via tail vein injection to pregnant *Pkm1*^+/−^ female mice bred with *Pkm1*^+/−^ males, and the embryos were harvested at E18.5 for subsequent analysis (Fig. 6d and Fig. S9b). Staining for Ki67 and pH3 showed that delivery of AAV9-cTnT-*Nfya* partially rescued the proliferation defects of KO cardiomyocytes (Fig. 6e, f). In addition, histological analysis of E18.5 hearts (Fig. 6g) revealed that AAV9-cTnT*-Nfya* treatment also partially restored the thickness of the compact myocardium which was reduced in KO embryos (Fig. 6h). No significant changes were observed in the trabecular zones across groups (Fig. 6h), implying that both PKM1 and NFYa predominantly affect the compact zone of the myocardium.

We also examined the effects of *Pkm1* knockdown and NFYa overexpression in human iPSC-derived cardiomyocytes (hiPSC-CMs). Consistent with our findings in NRVCs and mouse hearts, *Pkm1* knockdown in hiPSC-CMs impaired cardiomyocyte proliferation, which was significantly restored by NFYa overexpression (Fig. S9c). These results support the conservation of the PKM1-AMPK-NFYa axis in hiPSC-CMs, underscoring its potential relevance to human cardiac development and disease.

## DISCUSSION

By generating a *Pkm1*-specific premature termination mutation, we have made a mouse line with PKM1 ablation without compensatory PKM2 upregulation. Notably, while previous studies using conventional knockout strategies reported no overt cardiac phenotypes due to compensatory PKM2 splicing [20,21], our approach ablating PKM1 without triggering compensatory upregulation of PKM2, revealed the intrinsic requirement of PKM1 for maintaining the proliferative capacity of compact zone cardiomyocytes. Mechanistic study supports a working model that PKM1 maintains normal pyruvate flux and ATP production, preventing AMPK activation in the compact myocardium, which allows NFYa to promote expression of cell cycle genes, sustaining cardiac proliferation and cardiac development. Our work identified the PKM1-AMPK-NFYa axis in energetic regulation of cardiomyocyte proliferation during heart development, and may provide novel targets for energetic intervention for congenital heart diseases with cardiomyocyte proliferation defects.

Interestingly, the compact zone-specific defect in *Pkm1*-KO embryos is highly correlated with the spatiotemporal expression pattern of PKM1, which is enriched in the compact myocardium and increases during late gestation. This pattern aligns with the critical period of cardiac growth [33]. It has been well-documented that in late gestation, cardiomyocyte proliferation is enriched in the compact zone compared to the trabecular [34,35], and several theories, such as pro-proliferation signals from the epicardium [36,37], and enhanced maturation of the trabecular cardiomyocytes [38–40] have been proposed to explain this divergence. A few transcription factors, such as Hey2 [40] and Tbx20 [41,42] have been identified to be enriched in the compact zone myocardium which are important for their identity and proliferative capacity. Our work identified PKM1 as a metabolic enzyme that is enriched in the compact zone myocardium, where it facilitates cardiomyocyte proliferation through AMPK and NFYa. This finding provides a new layer of the regulatory network that underpins the proliferation of the compact zone cardiomyocytes, thereby highlighting a metabolic component in this regulation.

The stage-specific impact of PKM1 ablation on cardiomyocyte proliferation aligns with developmental shifts in cardiac energy management. While PKM1 is present in early embryonic hearts, its knockout only disrupts proliferation during late gestation, sparing early cardiac morphogenesis. This temporal specificity reflects a critical transition in energy prioritization: pre-E14.5 hearts tolerate PKM2-driven glycolysis despite its inherent inefficiency, as high AMPK activity in both WT and KO hearts (Fig. 3f) reveals an energy-stressed yet proliferation-permissive state. During this phase, the relatively little competing contraction demand allows energy allocation to favor growth even under suboptimal ATP production. The metabolic landscape transforms dramatically post-E14.5, as embryonic growth necessitates increased cardiac output. WT hearts resolve this challenge through PKM1-mediated metabolic enhancement: the sharp decline in AMPK phosphorylation reflects abundant ATP production matching combined needs for both contraction and proliferation. *Pkm1*-KO hearts, however, fail to provide sufficient ATP, resulting in sustained AMPK activation in late gestation KO hearts, forcing cardiomyocytes to prioritize contraction over proliferation. This energy triage mechanism explains the selective proliferation defect without overt contractile failure, as no peripheral edema was observed in *Pkm1*-KO embryos (data not shown), indicating that the basal ATP production suffices for vital contractions, while the energy surplus required for cell cycle progression becomes unavailable. Our findings extend previous observations of developmental AMPK dynamics [18] by demonstrating that PKM1 is essential for suppressing AMPK activity during metabolic maturation. The temporal correlation between PKM1 upregulation and AMPK inactivation suggests a ‘gear shift’ in late gestation cardiac metabolism. PKM2-dominated glycolysis, while adequate for early growth, cannot meet the compounded energy requirements of later stages. PKM1’s higher enzymatic efficiency enables pyruvate flux sufficient to both inactivate AMPK (through ATP surplus) and fuel mitochondrial ATP production, creating a permissive metabolic state for proliferation.

Previous studies have established NFYa as a crucial regulator of embryonic and perinatal cardiomyocyte proliferation [28,31], primarily through its interaction with metabolic and cell cycle genes. However, the upstream regulation of NFYa activity had remained largely unknown in cardiomyocytes. By linking NFYa activity to metabolic status, our findings demonstrated that the stability of NFYa is tightly regulated by energy availability through AMPK, establishing a novel regulatory mechanism of NFYa activity. Particularly, the identification of serine 325 as the phosphorylation site mediating NFYa degradation recognized a key regulatory molecular switch, as well as a potential target for modulating NFYa activity in metabolic disorders or developmental abnormalities. However, as a well-known kinase, whether AMPK directly phosphorylates NFYa or via an intermediate way remains unresolved. Future biochemical studies are essential to provide mechanistic insight into this critical interaction. The connection we established between metabolic status and cardiomyocyte proliferation through NFYa underscores the intricate interplay between metabolism and transcriptional regulation during cardiac development. Future studies could explore whether similar mechanisms operate in other high-energy-demand tissues, such as the brain and skeletal muscle, where PKM1 is also highly expressed.

The ability of AMPK to regulate NFYa stability highlights its dual role as a metabolic sensor and a regulator of cell fate decisions [29]. While AMPK activation is generally considered protective in conditions of energy stress, especially upon ischemic reperfusion injures [43], our findings suggest that AMPK is critical in allocating energy utilization during cardiac development, when fluctuation in energy production is not well tolerated. This multifaceted role of AMPK is further emphasized by clinical studies: gain-of-function mutations in *PRKAG2*, which encodes the AMPKγ2 regulatory subunit, are associated with congenital cardiac syndromes characterized by ventricular hypertrophy, conduction defects, and glycogen metabolism disorders [44–46]. Notably, severe AMPK-activating *PRKAG2* mutations such as the R531Q variant result in fatal neonatal cardiomyopathy with prenatal bradycardia and extreme cardiomegaly [46], mirroring the perinatal lethality and cardiac dysfunction observed in our *Pkm1*-KO mice. Interestingly, these patients exhibit massive glycogen accumulation and disrupted energy homeostasis—a phenotypic similarity to our model, where PKM1 deficiency triggers AMPK hyperactivation and metabolic dysregulation. These clinical observations, combined with our mechanistic data, underscore the importance of context-dependent regulation of AMPK and suggest that fine-tuning its activity could be a viable strategy for therapeutic intervention. For instance, while AMPK activation may be beneficial in conditions such as ischemia or heart failure to enhance energy efficiency, its inhibition could be advantageous in congenital heart diseases characterized by impaired cardiomyocyte proliferation.

The altered metabolomic profile in *Pkm1*-KO hearts suggests that the loss of PKM1 affects not only energy metabolism but also amino acid, nucleotide, and lipid metabolism, potentially contributing to the observed developmental defects. In fact, although NFYa overexpression partially rescued cardiomyocyte proliferation and ventricular wall thickness in *Pkm1*-KO hearts, AAV9-cTnT-*Nfya* did not significantly prolong the lifespan of *Pkm1*-KO mice (data not shown). On one hand, the extent of the cardiomyocyte proliferation recovery did not reach wild-type levels. On the other hand, the metabolic deficits associated with PKM1 loss, along with contributions from other downstream targets, likely synergize to drive neonatal cardiac dysfunction and compromised survival in *Pkm1*-KO mice. Future research could explore how PKM1 regulates material metabolism and if it plays a role in cardiac development.

Previous studies have reported the survivability of *Pkm1*-KO mice employing conditional knockout of *Pkm1*-specific exon [21], probably due to compensatory elevation of PKM2. In this study, a mutation was introduced into the *Pkm1*-specific exon, resulting in the nonsense-mediated decay (NMD) of the transcript. This led to the ablation of PKM1 and, maybe as secondary effect, downregulation of PKM2 as we observed. Consequently, the phenotype may not rest entirely on removal of PKM1 alone but also involves downregulation of PKM2. Thus, our model is not without limitation just like the previous study, where the availability of *Pkm1*-KO mice was probably not lying in the dispensability of PKM1 but the redundant elevation of PKM2. Here, by employing a distinct model, we presented an alternative outcome of *Pkm1*-KO mice rather than contradicting prior findings.

Finally, the translational potential of our findings should be explored. The identification of the PKM1-AMPK-NFYa axis provides a clear target for therapeutic intervention, but the development of effective strategies to modulate this pathway *in vivo* remains a challenge. While AMPK inhibitors show promise in rescuing proliferation defects in our study, their systemic effects and potential off-target impacts need to be carefully evaluated. Similarly, the delivery of NFYa via AAV9 vectors raises questions about the long-term safety and efficacy of such approaches. In light of these findings, future studies could explore small-molecule activators of PKM1 to enhance pyruvate flux and mitochondrial ATP synthesis in cardiomyocytes. Additionally, selective AMPK modulators may help fine-tune this kinase’s dual role in energy sensing and developmental regulation. Furthermore, strategies aimed at stabilizing NFYa—such as peptide mimetics or small molecules that prevent its degradation—may provide a more targeted and practical therapeutic route. These interventions could be particularly valuable in congenital heart diseases involving energy imbalance and proliferative failure of cardiomyocytes.

In conclusion, our study establishes PKM1 as a critical regulator of cardiomyocyte proliferation and cardiac development, acting through an energy-dependent mechanism that links mitochondrial function to transcriptional control. By uncovering the PKM1-AMPK-NFYa axis, we provide a novel framework for understanding the metabolic regulation of heart development and its implications for congenital heart diseases. These findings highlight the potential of targeting metabolic pathways to enhance cardiomyocyte proliferation and repair, and future research building on these insights could pave the way for innovative strategies to address the growing burden of congenital cardiac disorders.

## MATERIALS AND METHODS

All animal experiments were approved by the Biological Research Ethics Committee of Tongji University. Detailed materials and methods are available in the Supplementary data.

## Supplementary Material

nwaf408_Supplemental_Files

## Data Availability

The RNA-Seq data generated in this study have been deposited in the Gene Expression Omnibus (GEO) with accession number GSE290043. Publicly available datasets used in this study are as follows: GSE130699 (Fig. S4e, f), GSE232961 (Fig. 4b), and GSE232960 (Fig. 4c, d).

## References

[1] Bornstein MR, Tian R, Arany Z. Human cardiac metabolism. Cell Metab 2024; 36: 1456–81. 10.1016/j.cmet.2024.06.003 38959861 PMC11290709

[2] Cohen P, Kajimura S. The cellular and functional complexity of thermogenic fat. Nat Rev Mol Cell Biol 2021; 22: 393–409. 10.1038/s41580-021-00350-0 33758402 PMC8159882

[3] Belanger M, Allaman I, Magistretti PJ. Brain energy metabolism: focus on astrocyte-neuron metabolic cooperation. Cell Metab 2011; 14: 724–38. 10.1016/j.cmet.2011.08.016 22152301

[4] Wculek SK, Dunphy G, Heras-Murillo I et al. Metabolism of tissue macrophages in homeostasis and pathology. Cell Mol Immunol 2022; 19: 384–408. 10.1038/s41423-021-00791-9 34876704 PMC8891297

[5] Zhang J, Zhao J, Dahan P et al. Metabolism in pluripotent stem cells and early mammalian development. Cell Metab 2018; 27: 332–8. 10.1016/j.cmet.2018.01.008 29414683

[6] Amorim JA, Coppotelli G, Rolo AP et al. Mitochondrial and metabolic dysfunction in ageing and age-related diseases. Nat Rev Endocrinol 2022; 18: 243–58. 10.1038/s41574-021-00626-7 35145250 PMC9059418

[7] Nakamura M, Sadoshima J. Mechanisms of physiological and pathological cardiac hypertrophy. Nat Rev Cardiol 2018; 15: 387–407. 10.1038/s41569-018-0007-y 29674714

[8] Kolwicz SC Jr., Purohit S, Tian R. Cardiac metabolism and its interactions with contraction, growth, and survival of cardiomyocytes. Circ Res 2013; 113: 603–16. 10.1161/CIRCRESAHA.113.302095 23948585 PMC3845521

[9] Dayton TL, Jacks T, Vander Heiden MG. PKM2, cancer metabolism, and the road ahead. EMBO Rep 2016; 17: 1721–30. 10.15252/embr.201643300 27856534 PMC5283597

[10] Chen M, David CJ, Manley JL. Concentration-dependent control of pyruvate kinase M mutually exclusive splicing by hnRNP proteins. Nat Struct Mol Biol 2012; 19: 346–54. 10.1038/nsmb.2219 22307054 PMC3698866

[11] David CJ, Chen M, Assanah M et al. HnRNP proteins controlled by c-Myc deregulate pyruvate kinase mRNA splicing in cancer. Nature 2010; 463: 364–8. 10.1038/nature08697 20010808 PMC2950088

[12] Jia Y, Mao C, Ma Z et al. PHB2 maintains the contractile phenotype of VSMCs by counteracting PKM2 splicing. Circ Res 2022; 131: 807–24. 10.1161/CIRCRESAHA.122.321005 36200440

[13] Christofk HR, Vander Heiden MG, Harris MH et al. The M2 splice isoform of pyruvate kinase is important for cancer metabolism and tumour growth. Nature 2008; 452: 230–3. 10.1038/nature06734 18337823

[14] Yang W, Xia Y, Hawke D et al. PKM2 phosphorylates histone H3 and promotes gene transcription and tumorigenesis. Cell 2012; 150: 685–96. 10.1016/j.cell.2012.07.018 22901803 PMC3431020

[15] Magadum A, Singh N, Kurian AA et al. Pkm2 regulates cardiomyocyte cell cycle and promotes cardiac regeneration. Circulation 2020; 141: 1249–65. 10.1161/CIRCULATIONAHA.119.043067 32078387 PMC7241614

[16] Nakano H, Nakano A. The role of metabolism in cardiac development. Curr Top Dev Biol 2024; 156: 201–43. 38556424 10.1016/bs.ctdb.2024.01.005

[17] Subramanian A, Tamayo P, Mootha VK et al. Gene set enrichment analysis: a knowledge-based approach for interpreting genome-wide expression profiles. Proc Natl Acad Sci USA 2005; 102: 15545–50. 10.1073/pnas.0506580102 16199517 PMC1239896

[18] Xu M, Yao J, Shi Y et al. The SRCAP chromatin remodeling complex promotes oxidative metabolism during prenatal heart development. Development 2021; 148: dev199026. 10.1242/dev.199026 33913477

[19] Cardoso AC, Lam NT, Savla JJ et al. Mitochondrial substrate utilization regulates cardiomyocyte cell cycle progression. Nat Metab 2020; 2: 167–78. 10.1038/s42255-020-0169-x 32617517 PMC7331943

[20] Morita M, Sato T, Nomura M et al. PKM1 confers metabolic advantages and promotes cell-autonomous tumor cell growth. Cancer Cell 2018; 33: 355–67. 10.1016/j.ccell.2018.02.004 29533781

[21] Li Q, Li C, Elnwasany A et al. PKM1 exerts critical roles in cardiac remodeling under pressure overload in the heart. Circulation 2021; 144: 712–27. 10.1161/CIRCULATIONAHA.121.054885 34102853 PMC8405569

[22] Chen M, Zhang J, Manley JL. Turning on a fuel switch of cancer: hnRNP proteins regulate alternative splicing of pyruvate kinase mRNA. Cancer Res 2010; 70: 8977–80. 10.1158/0008-5472.CAN-10-2513 20978194 PMC2982937

[23] Lv L, Xu YP, Zhao D et al. Mitogenic and oncogenic stimulation of K433 acetylation promotes PKM2 protein kinase activity and nuclear localization. Mol Cell 2013; 52: 340–52. 10.1016/j.molcel.2013.09.004 24120661 PMC4183148

[24] Kuranaga Y, Sugito N, Shinohara H et al. SRSF3, a splicer of the PKM gene, regulates cell growth and maintenance of cancer-specific energy metabolism in colon cancer cells. Int J Mol Sci 2018; 19: 3012. 10.3390/ijms19103012 30279379 PMC6213643

[25] Israelsen WJ, Dayton TL, Davidson SM et al. PKM2 isoform-specific deletion reveals a differential requirement for pyruvate kinase in tumor cells. Cell 2013; 155: 397–409. 10.1016/j.cell.2013.09.025 24120138 PMC3850755

[26] Dayton TL, Gocheva V, Miller KM et al. Germline loss of PKM2 promotes metabolic distress and hepatocellular carcinoma. Genes Dev 2016; 30: 1020–33. 10.1101/gad.278549.116 27125672 PMC4863734

[27] Davidson SM, Schmidt DR, Heyman JE et al. Pyruvate kinase M1 suppresses development and progression of prostate adenocarcinoma. Cancer Res 2022; 82: 2403–16. 10.1158/0008-5472.CAN-21-2352 35584006 PMC9256808

[28] Cui M, Wang Z, Chen K et al. Dynamic transcriptional responses to injury of regenerative and non-regenerative cardiomyocytes revealed by single-nucleus RNA sequencing. Dev Cell 2020; 53: 102–116.e8. 10.1016/j.devcel.2020.02.019 32220304 PMC7365574

[29] Lin SC, Hardie DG. AMPK: sensing glucose as well as cellular energy status. Cell Metab 2018; 27: 299–313. 10.1016/j.cmet.2017.10.009 29153408

[30] Hawley SA, Davison M, Woods A et al. Characterization of the AMP-activated protein kinase kinase from rat liver and identification of threonine 172 as the major site at which it phosphorylates AMP-activated protein kinase. J Biol Chem 1996; 271: 27879–87. 10.1074/jbc.271.44.27879 8910387

[31] Cui M, Bezprozvannaya S, Hao T et al. Transcription factor NFYa controls cardiomyocyte metabolism and proliferation during mouse fetal heart development. Dev Cell 2023; 58: 2867–80. 10.1016/j.devcel.2023.10.012 37972593 PMC11000264

[32] Hornbeck PV, Zhang B, Murray B et al. PhosphoSitePlus, 2014: mutations, PTMs and recalibrations. Nucleic Acids Res 2015; 43: D512–20. 10.1093/nar/gku1267 25514926 PMC4383998

[33] Samsa LA, Yang B, Liu J. Embryonic cardiac chamber maturation: trabeculation, conduction, and cardiomyocyte proliferation. Am J Med Genet C Semin Med Genet 2013; 163C: 157–68. 10.1002/ajmg.c.31366 23720419 PMC3723796

[34] Sedmera D, Thompson RP. Myocyte proliferation in the developing heart. Dev Dyn 2011; 240: 1322–34. 10.1002/dvdy.22650 21538685 PMC3271704

[35] Sedmera D, Reckova M, DeAlmeida A et al. Spatiotemporal pattern of commitment to slowed proliferation in the embryonic mouse heart indicates progressive differentiation of the cardiac conduction system. Anat Rec 2003; 274: 773–7. 10.1002/ar.a.10085 12923887

[36] Merki E, Zamora M, Raya A et al. Epicardial retinoid X receptor alpha is required for myocardial growth and coronary artery formation. Proc Natl Acad Sci USA 2005; 102: 18455–60. 10.1073/pnas.0504343102 16352730 PMC1317903

[37] Stuckmann I, Evans S, Lassar AB. Erythropoietin and retinoic acid, secreted from the epicardium, are required for cardiac myocyte proliferation. Dev Biol 2003; 255: 334–49. 10.1016/S0012-1606(02)00078-7 12648494

[38] Zhang W, Chen H, Qu X et al. Molecular mechanism of ventricular trabeculation/compaction and the pathogenesis of the left ventricular noncompaction cardiomyopathy (LVNC). Am J Med Genet C Semin Med Genet 2013; 163C: 144–56. 10.1002/ajmg.c.31369 23843320 PMC3725649

[39] Wu M . Mechanisms of trabecular formation and specification during cardiogenesis. Pediatr Cardiol 2018; 39: 1082–9. 10.1007/s00246-018-1868-x 29594501 PMC6164162

[40] Li J, Miao L, Shieh D et al. Single-cell lineage tracing reveals that oriented cell division contributes to trabecular morphogenesis and regional specification. Cell Rep 2016; 15: 158–70. 10.1016/j.celrep.2016.03.012 27052172 PMC4826812

[41] Zhang W, Chen H, Wang Y et al. Tbx20 transcription factor is a downstream mediator for bone morphogenetic protein-10 in regulating cardiac ventricular wall development and function. J Biol Chem 2011; 286: 36820–9. 10.1074/jbc.M111.279679 21890625 PMC3196085

[42] Chakraborty S, Sengupta A, Yutzey KE. Tbx20 promotes cardiomyocyte proliferation and persistence of fetal characteristics in adult mouse hearts. J Mol Cell Cardiol 2013; 62: 203–13. 10.1016/j.yjmcc.2013.05.018 23751911

[43] Cai J, Chen X, Liu X et al. AMPK: the key to ischemia-reperfusion injury. J Cell Physiol 2022; 237: 4079–96. 10.1002/jcp.30875 36134582

[44] Arad M, Benson DW, Perez-Atayde AR et al. Constitutively active AMP kinase mutations cause glycogen storage disease mimicking hypertrophic cardiomyopathy. J Clin Invest 2002; 109: 357–62. 10.1172/JCI0214571 11827995 PMC150860

[45] Zhan Y, Sun X, Li B et al. Establishment of a PRKAG2 cardiac syndrome disease model and mechanism study using human induced pluripotent stem cells. J Mol Cell Cardiol 2018; 117: 49–61. 10.1016/j.yjmcc.2018.02.007 29452156

[46] Burwinkel B, Scott JW, Bührer C et al. Fatal congenital heart glycogenosis caused by a recurrent activating R531Q mutation in the γ2-subunit of AMP-activated protein kinase (*PRKAG2*), not by phosphorylase kinase deficiency. Am J Hum Genet 2005; 76: 1034–49. 10.1086/430840 15877279 PMC1196441

