# Supplementary material for: PKM1 is required for embryonic cardiomyocyte proliferation through energetic regulation of NFYa stability
Source: Natl Sci Rev. 2025 Sep 25;13(1):nwaf408. doi: 10.1093/nsr/nwaf408 (PMC12796809; doi:10.1093/nsr/nwaf408)
Supplement: nwaf408_Supplemental_Files [file nwaf408_supplemental_files.zip › Supplementary data-250911.docx]

**Supplementary data for**

**Pkm1 is required for embryonic cardiomyocyte proliferation through energetic regulation of NFYa stability**

Dandan Zhang^1, 6^, Yansong Tang^1, 6^, Wen Ye^1, 6^, Danli Yang^5^, Shengtang Qin^5^, Juntao Liu^1^, Nan Su^1^, Rongrong Huang^4^, Guangzheng Shi^4^, Dachun Xu^4^, Xiaochen Kou^3^, Yanhong Zhao^3^, Hong Wang^2^, Shaorong Gao^2, 3^*, Ke Wei^1,^ *, Lan Kang^1, 3^*

^1^ Institute for Regenerative Medicine, Shanghai East Hospital, Shanghai Institute of Stem Cell Research and Clinical Translation, Shanghai Key Laboratory of Signaling and Disease Research, Frontier Science Center for Stem Cell Research, School of Life Sciences and Technology, Tongji University, Shanghai 200092, China

^2^ Clinical and Translation Research Center of Shanghai First Maternity & Infant Hospital, School of Life Sciences and Technology, Tongji University, Shanghai 200092, China

^3^ Frontier Science Center for Stem Cell Research, Tongji University, Shanghai 200092, China

^4^ Department of Cardiology, Clinical Research Unit, Shanghai Tenth People’s Hospital, Tongji University School of Medicine, Shanghai 200092, China

^5^ Institute of Cancer Stem Cell, Dalian Medical University, Dalian 116044, China

^6^ These authors contributed equally: Dandan Zhang, Yansong Tang, Wen Ye.

^*^ Corresponding authors:

Lan Kang, PhD, Email: kanglan@tongji.edu.cn, Tel: 86-21-65982276

Ke Wei, PhD, Email: kewei@tongji.edu.cn, Tel: 86-21-65981041

Shaorong Gao, PhD, Email: gaoshaorong@tongji.edu.cn, Tel: 86-21-65985182

**This file includes:**

Materials and Methods, Supplementary Figures S1 to S9, Supplementary Tables S1 to S3.

**Materials and Methods**

**Animal use and care**

C57BL/6J, DBA/2, and ICR mice were purchased from Charles River (China). The specific pathogen-free (SPF) grade mice, including C57BL/6J, DBA/2, ICR and BDF1 mice, were housed in the animal facility at Tongji University, Shanghai, China. All the mice had free access to food and water, and were housed on a 12 h light/dark cycle.

All animal maintenance and experimental procedures were performed according to the Tongji University guide for the use of laboratory animals. All animal experiments were approved by the Biological Research Ethics Committee of Tongji University.

The BDF1 hybrid mice were obtained by mating male DBA/2 mice with female C57BL/6J mice. BDF1 female mice (8-10 weeks old) were superovulated via injection with 5 IU of pregnant mare serum gonadotropin (PMSG), followed by injection of 6 IU of human chorionic gonadotropin (hCG) (San-Sheng Pharmaceutical Co., Ltd.) 48 h later. The superovulated female mice were mated with BDF1 male mice and zygotes were collected 20 h later. Cas9 mRNA (100 ng/μL) and sgRNAs (50 ng/μL each) were injected into zygotes by a Piezo-driven micromanipulator and then cultured in G-1 PLUS medium (Vitrolife) at 37°C in humidified atmosphere of 5% CO_2_. At ~28 h later, 2-cell embryos were transferred into oviducts of pseudo-pregnant ICR mothers.

mMESSAGE mMACHINE T7 Ultra Kit (Ambion, Thermo Fisher Scientific, AM1345) and MEGAshortscrip T7 Transcription Kit (Ambion, Thermo Fisher Scientific, AM1354) were used for the synthesis of Cas9 mRNA and sgRNAs, respectively. All Cas9 mRNA and sgRNAs were purified according to the standard protocol by phenol:chloroform (Sigma) extraction and ethanol precipitation, and then dissolved in DNase/RNase-free water.

For genotyping, tail tips and toes from mice were lysed in lysis buffer (25 mM NaOH, 0.2 mM EDTA) and genomic DNA was extracted as template for PCR. With the two primers, it is possible to amplify specific WT (110 bp) and mutant (84 bp) DNA fragments. sgRNA and primer sequences are listed in Table S1.

Potential off-target loci of the *Pkm1* sgRNA were predicted using the CRISPOR web tool (https://crispor.gi.ucsc.edu/). The top 15 predicted off-target sites were selected for experimental validation. Genomic regions spanning these candidate loci were amplified by PCR. Amplified products were subsequently analyzed by Sanger sequencing and TIDE to detect potential CRISPR/Cas9-mediated modifications. TIDE analysis was performed using Sanger sequencing trace files and the web-based TIDE tool (<https://tide.nki.nl/>), with wild-type mice serving as the control reference. Detailed information on predicted off-target loci and sequencing results are provided in Table S2.

**Neonatal rat ventricular cardiomyocytes (NRVCs) isolation and treatment**

NRVCs were isolated from 1-day-old Sprague-Dawley rats (obtained from the Shanghai Laboratory Animal Center, CAS) using a 0.25% trypsin (Gibco) dissociation method performed at 4°C overnight. On the subsequent day, the heart tissues were further digested with 0.1% collagenase type II (Worthington) to release the cells. Isolated cardiomyocytes were cultured in Dulbecco's Modified Eagle Medium (DMEM, Gibco) supplemented with 10% fetal bovine serum (FBS, Gibco) and penicillin/streptomycin. After initial culture, the medium was replaced with a specialized cardiomyocyte culture medium composed of DMEM and F12 (1:1) supplemented with 0.25% FBS, 3 mM sodium pyruvate, 0.1 mM vitamin C, and 2 mM L-glutamine.

Gene knockdown experiments in NRVCs were performed using Lipofectamine 2000 transfection reagent (Invitrogen), following the manufacturer’s instructions. Post-transfection, cells were harvested at two time points: 24 h for RNA extraction and 48 h for whole-cell protein extraction. Cells were washed in phosphate-buffered saline (PBS) prior to these procedures to remove any residual media components.

AAV9 viral infection of NRVCs was completed in suspension immediately after cell isolation. NRVCs were infected with the AAV9 virus at a multiplicity of infection (MOI) of 100000 for 40 h. NRVCs were fixed for staining or lysed for protein extraction at 48 h after removing virus from cardiomyocytes.

**Histology**

Heart, lung and diaphragm tissues were harvested, washed twice in PBS (Servicebio, China) and fixed in 4% paraformaldehyde (PFA) (Servicebio, China) overnight. Subsequently, the samples were dehydrated stepwise through an ethanol series (70%, 80%, 90% and 100% ethanol), embedded in paraffin and serially sectioned at 5-μm thickness. Standard hematoxylin and eosin staining were performed on these sections to evaluate histopathology.

**Fluorescence staining**

Hearts were harvested, washed twice in PBS and fixed in 4% PFA at 4°C overnight. The next day, hearts were washed with PBS and incubated in 30% sucrose at 4°C overnight. Next, hearts were put in OCT (SAKURA, 4583), frozen and stored at -80°C. The heart blocks were sectioned at 6-7µm using cryostat. NRVCs were fixed with 4% PFA at 4°C overnight.

For immunofluorescence analysis, heart sections and fixed cells were permeabilized with 0.5% Triton-X100/PBS for 15 minutes at room temperature. Samples were blocked with 3% bovine serum albumin (BSA) (MP Biomedicals) in PBS at room temperature for 1h and were then incubated with primary antibodies diluted in 3% BSA in PBS overnight at 4°C. After washing three times with PBS, the samples were incubated with the appropriate secondary antibodies (Thermo Fisher Scientific) at room temperature in the dark for 1h. The nuclei were stained with 4’,6-diamidino-2-phenylindole (DAPI) (Invitrogen, D3571) at 1 μg/mL. A ZEISS LSM 880 microscope was used for imaging. Signal intensity statistics were performed using ZEISS processing software. Antibodies used in this study are shown in Table S3.

For wheat germ agglutinin (WGA) staining, frozen sections were rehydrated, permeabilized, and blocked with 3% BSA at room temperature. Cardiomyocyte membrane was stained with Alexa Fluor 594-conjugated WGA (10 μg/mL, Thermo Fisher Scientific, #W11262) for 30 minutes and washed three times in PBS. Then, slides were incubated with DAPI.

The TUNEL (TdT-mediated dUTP Nick-End Labeling) assay was performed with the DeadEnd Fluorometric TUNEL System (Promega, G3250) according to the instructions of the manufacturer. Then, slides were incubated with primary antibodies, secondary antibodies, and DAPI as described above.

**Reverse transcription and quantitative Real Time-PCR (RT-qPCR)**

Total RNA was extracted using TRIzol reagent (TaKaRa) and reverse transcribed using an All-In-One RT Master Mix (ABM, G490) according to the manufacturer’s recommendations. qPCR was carried out using TB Green Premix Ex Taq II (TaKaRa) and monitored by 7500 Fast Real-Time PCR System, and three technical replicates were performed for each sample. qPCR primers for tested genes are listed in Table S1.

**Western blot**

Total proteins were extracted from mouse tissues or NRVCs using RIPA lysis buffer (Beyotime, P0013B) containing the protease inhibitor cocktail (Roche, 04693132001). The lysates were boiled to 95°C for 10 minutes in Omni-Easy Protein Sample Loading Buffer (EpiZyme). Proteins were then separated by 10% SDS/PAGE and electro-transferred onto PVDF membranes (Millipore, IPVH00010). The membranes were then blocked with 5% skim milk in TBST (EpiZyme) and incubated with primary antibodies (1:1000) overnight at 4°C. After washing three times for 10 minutes with TBST, membranes were incubated with corresponding HRP-conjugated secondary antibodies (1:2000) diluted in blocking buffer for 1 h at room temperature. The signals were measured using ECL reagents (Thermo Scientific, USA) and were visualized by the ChemiDoc MP imaging system (Bio-Rad).

**Metabolomics analysis**

Metabolomics analysis was conducted by Shanghai Applied Protein Technology. Briefly, metabolites from heart samples were extracted through lysis, sonication, and centrifugation. Untargeted metabolomics of polar metabolites was performed, and extracts were analyzed using a quadrupole time-of-flight mass spectrometer (SCIEX TripleTOF 6600) coupled to hydrophilic interaction chromatography via electrospray ionization. The mass spectrometer was operated in both negative ion and positive ion modes.

The raw MS data were converted to MzXML files using ProteoWizard MSConvert before data processing using XCMS software. In the extracted ion features, only variables having more than 50% of the nonzero measurement values in at least one group were retained. After normalization to total peak intensity, differentially expressed metabolite analysis was performed, and metabolites with log2 (fold change) > 1 and *P* values < 0.05 were considered differentially expressed metabolites. Differentially expressed metabolites were then uploaded to MetaboAnalyst 5.0 for KEGG pathway annotation. Pathways with *P* values < 0.05 were considered significantly changed pathways.

**Seahorse assay**

Mitochondrial oxygen consumption rates (OCR) were determined using the Seahorse XF96 Analyzer (Agilent). NRVCs were seeded onto a Seahorse XF96 Cell Culture Microplate (Agilent) at a density of 4×10^4^ cells per well and treated with siRNA. The following drugs were injected at the final concentrations given: oligomycin (1 μM), carbonyl cyanide 4-(trifluoromethoxy) phenylhydrazone (FCCP, 2 μM), rotenone (1 μM) and antimycin A (1 μM).

**Determination of ATP concentration**

Mouse hearts were freshly isolated from E18.5 embryos and immediately processed for ATP detection. ATP levels of hearts were determined using an Enhanced ATP Assay Kit (Beyotime, S0027) following the manufacturer’s instructions.

**AAV9 preparation and injection**

AAV9-cTnT-*Nfya* viruses were customer prepared by Shanghai Taitool Bioscience, Co., Ltd. For each D12.5 pregnant mouse, 100 μL of PBS containing 3×10^11^ GC AAV9 viruses was injected through tail vein. Six days after injection, the E18.5 mouse hearts were harvested for analysis.

**Ribosomal RNA-free total RNA-Seq**

Total RNA was extracted using TRIzol reagent (TaKaRa). Three biological replicates for each genotype were prepared, and 1 μg RNA per sample was subjected to rRNA elimination and RNA-seq library generation using KAPA RiboErase Kit and KAPA RNA HyperPrep Kit following manufacturer's instructions. Libraries were sequenced on the Illumina NovaSeq 6000 platform with paired ends and 150-bp read lengths (Nanjing Jiangbei New Area Biophamaceutical Public Service Platform).

**RNA-Seq data analysis**

Clean sequencing reads were aligned to the reference genome using the Hisat2 alignment tool, and transcript assembly was performed using StringTie. Differential gene expression analysis was conducted using the edgeR package, which processes the expression matrix to identify statistically significant changes in gene expression. Gene Ontology (GO) and Kyoto Encyclopedia of Genes and Genomes (KEGG) enrichment analyses of the differentially expressed genes (DEGs) were performed using the ClusterProfiler. GO terms with adjusted *P*-values (FDR) less than 0.05 were considered significantly enriched.

**Single-Cell RNA Sequencing (scRNA-Seq) data analysis**

**Data collection and preprocessing:** Single-cell RNA sequencing data were sourced from previously published datasets and analyzed using the Seurat package in R. Initial quality control steps involved filtering cells based on mitochondrial gene content, unique gene counts, and overall expression thresholds to ensure data integrity. Dimensionality reduction was achieved through principal component analysis (PCA).

**Clustering and visualization:** Cell clustering was carried out using the Louvain algorithm to identify discrete cellular subpopulations. Visualization of clusters was achieved through Uniform Manifold Approximation and Projection (UMAP), implemented using the SCP package in R for intuitive interpretation of the high-dimensional data.

**Statistics analysis**

Data analyses were performed using GraphPad Prism software (version 10.1.2). Results are expressed as mean ± SEM. Unpaired t-test was used to determine significance for two-group comparison, and one-way ANOVA followed by multiple comparisons was used to determine significance where three or more groups were included. Differences at the level of *P* < 0.05 were considered statistically significant. Number of replicates are noted for each experiment in the respective figure legends. Unless otherwise indicated, all replicates (*n*) refer to biologically independent samples.


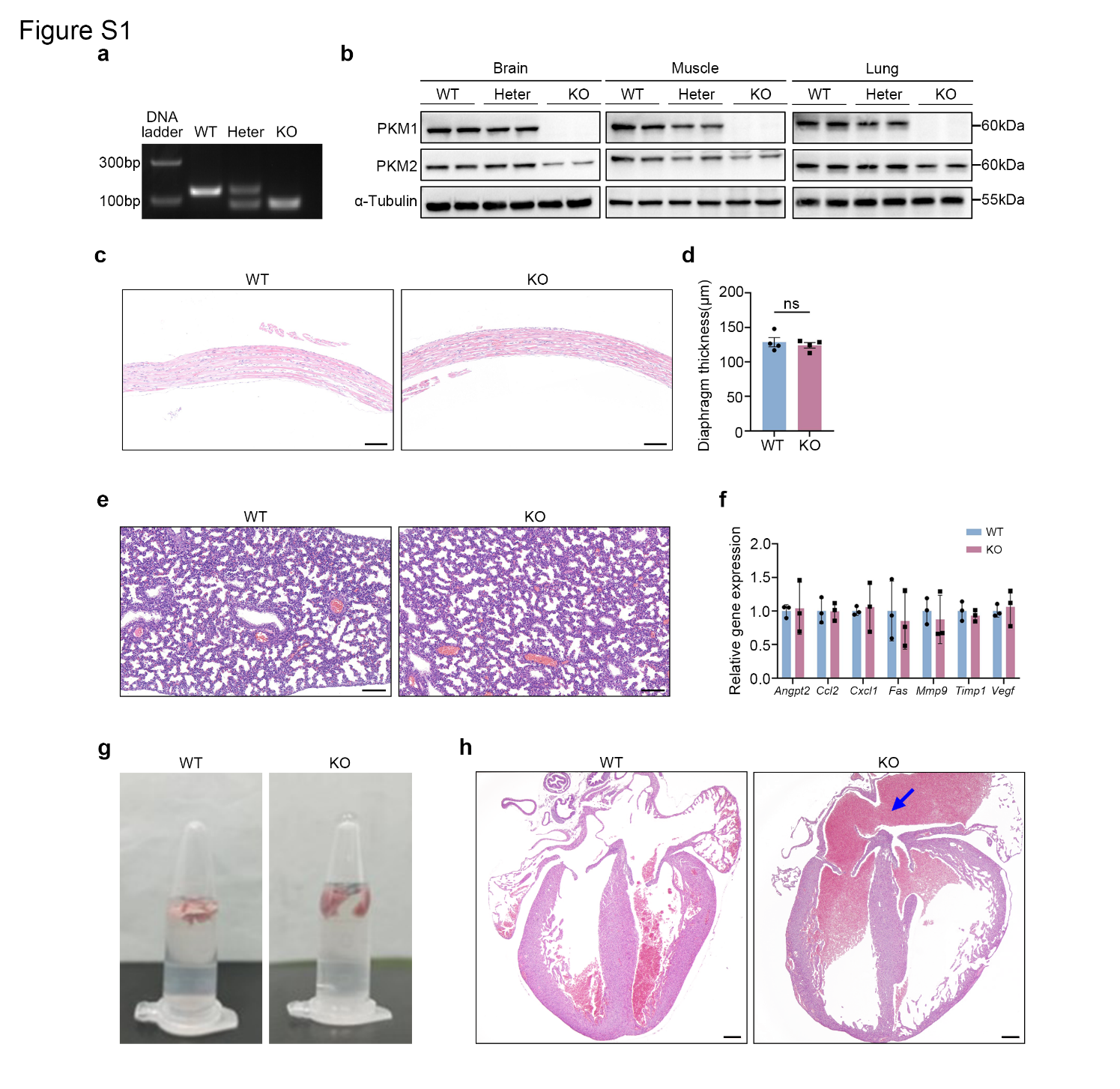


**Supplementary Fig. S1 Identification and cardiopulmonary function assessment of *Pkm1*-KO embryos.**

(a) PCR genotyping results from germline mice of the indicated genotypes using primers listed in Table S1. Genotyping by amplifying the specific wild-type (WT) (110 bp) and mutant (84 bp) DNA fragments. KO, knockout. Heter, heterozygous. (b) Western blot analysis of PKM1, PKM2 protein levels in tissues from WT, Heter and KO embryos at embryonic days (E) 18.5. (c) Hematoxylin-eosin (H&E) staining of E18.5 WT and KO diaphragms. Scale bars, 100 µm. (d) Quantification of diaphragm thicknesses of E18.5 WT and KO mice (*n* = 4 embryos per group). (e) H&E staining of E18.5 WT and KO lungs. Scale bars, 100 µm. (f) Stress markers expression in E18.5 WT and KO lungs (*n* = 3 embryos per group). All genes showed no significant difference in expression between two groups. (g) Representative photograph of lungs of postnatal day 0 (P0) mice. (h) H&E staining of E18.5 WT and KO hearts. Blue arrow indicates atrial septal defect (ASD). Scale bars, 200 µm. Two-tailed unpaired Student’s t test was performed (d, f). All quantitative data are expressed as mean ± SEM, **p* < 0.05, ns, not significant.


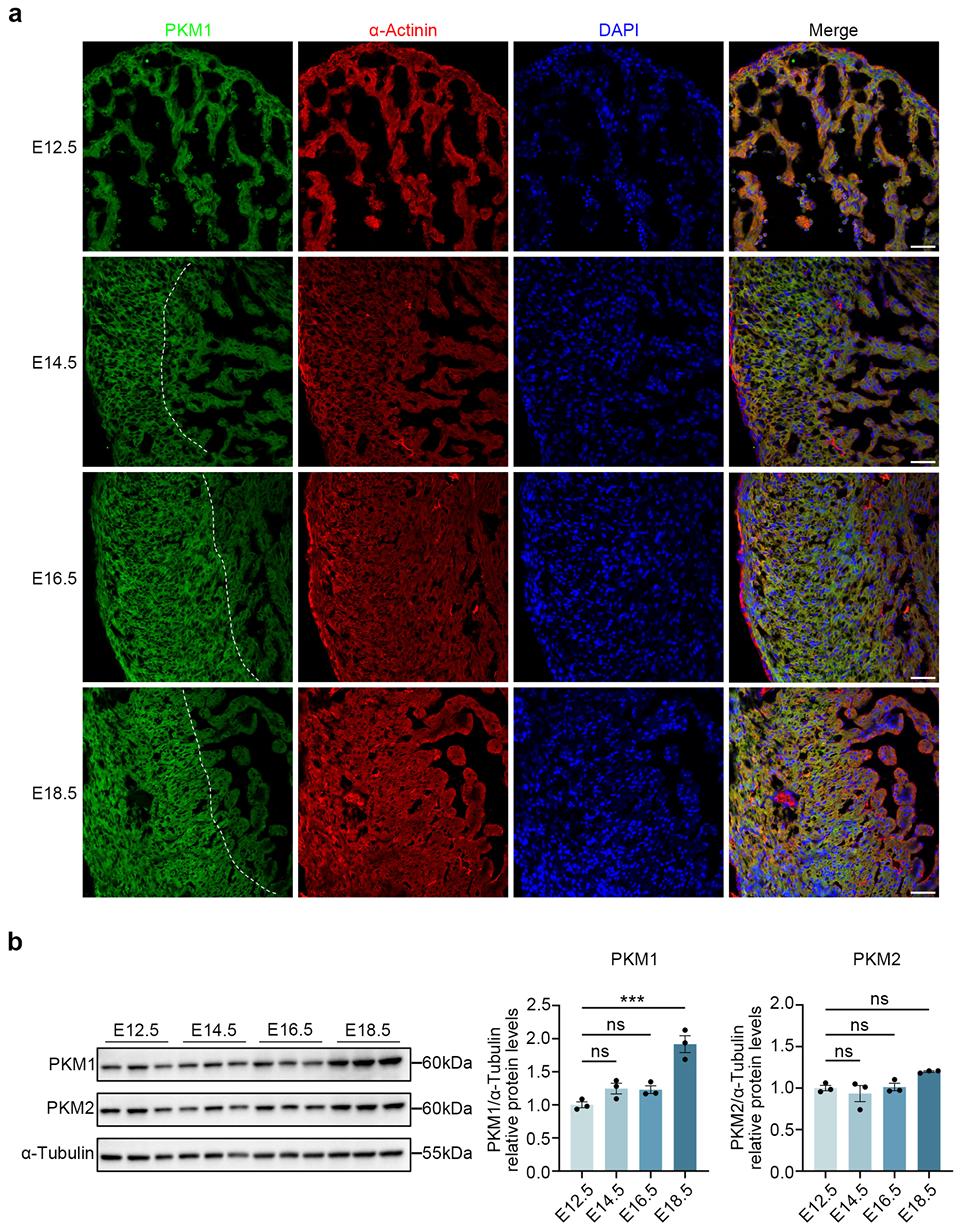


**Supplementary Fig. S2 Expression pattern of PKM1 in the embryonic mouse heart.**

(a) Representative images of PKM1 expression in mouse hearts at E12.5, E14.5, E16.5, and E18.5. Scale bars, 50 µm. (b) Western blot analysis and quantification of PKM1, PKM2 protein levels in mouse hearts at E12.5, E14.5, E16.5, and E18.5 (*n* = 3 hearts per group). One-way ANOVA, Tukey's Multiple Comparison Test was performed (b). All quantitative data are expressed as mean ± SEM, **p* < 0.05, ***p* < 0.01, ****p* < 0.001, ns, not significant.


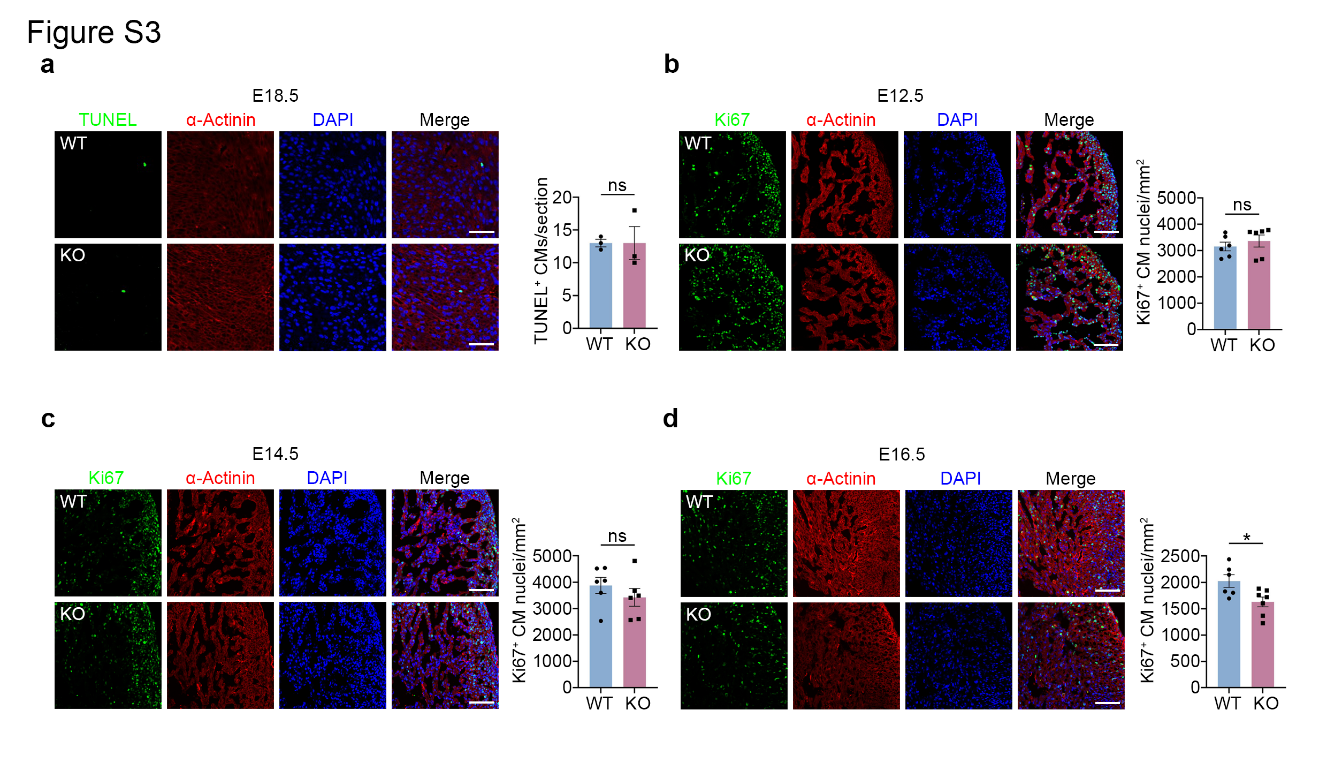


**Supplementary Fig. S3 Apoptosis and proliferation of cardiomyocytes in *Pkm1*-KO embryos at different developmental stages.**

(a) Representative images of terminal deoxynucleotidyl transferase dUTP nick end labeling (TUNEL)staining and quantification of cell death of cardiomyocytes in E18.5 WT and KO hearts (*n* = 3 hearts per group). Scale bars, 50 µm. (b) Representative images of Ki67 staining and quantification of Ki67^+^ nuclei per mm^2^ area of WT and KO hearts at E12.5 (*n* = 6 hearts per group). Scale bars, 100 µm. (c) Representative images of Ki67 staining and quantification of Ki67^+^ nuclei per mm^2^ area of WT and KO hearts at E14.5 (*n* = 6 hearts per group). Scale bars, 100 µm. (d) Representative images of Ki67 staining and quantification of Ki67^+^ nuclei per mm^2^ area of WT and KO hearts at E16.5 (*n* = 6 hearts for WT, 7 hearts for KO). Scale bars, 100 µm. Two-tailed unpaired Student’s t test was performed (a-d). All quantitative data are expressed as mean ± SEM, **p* < 0.05, ns, not significant.


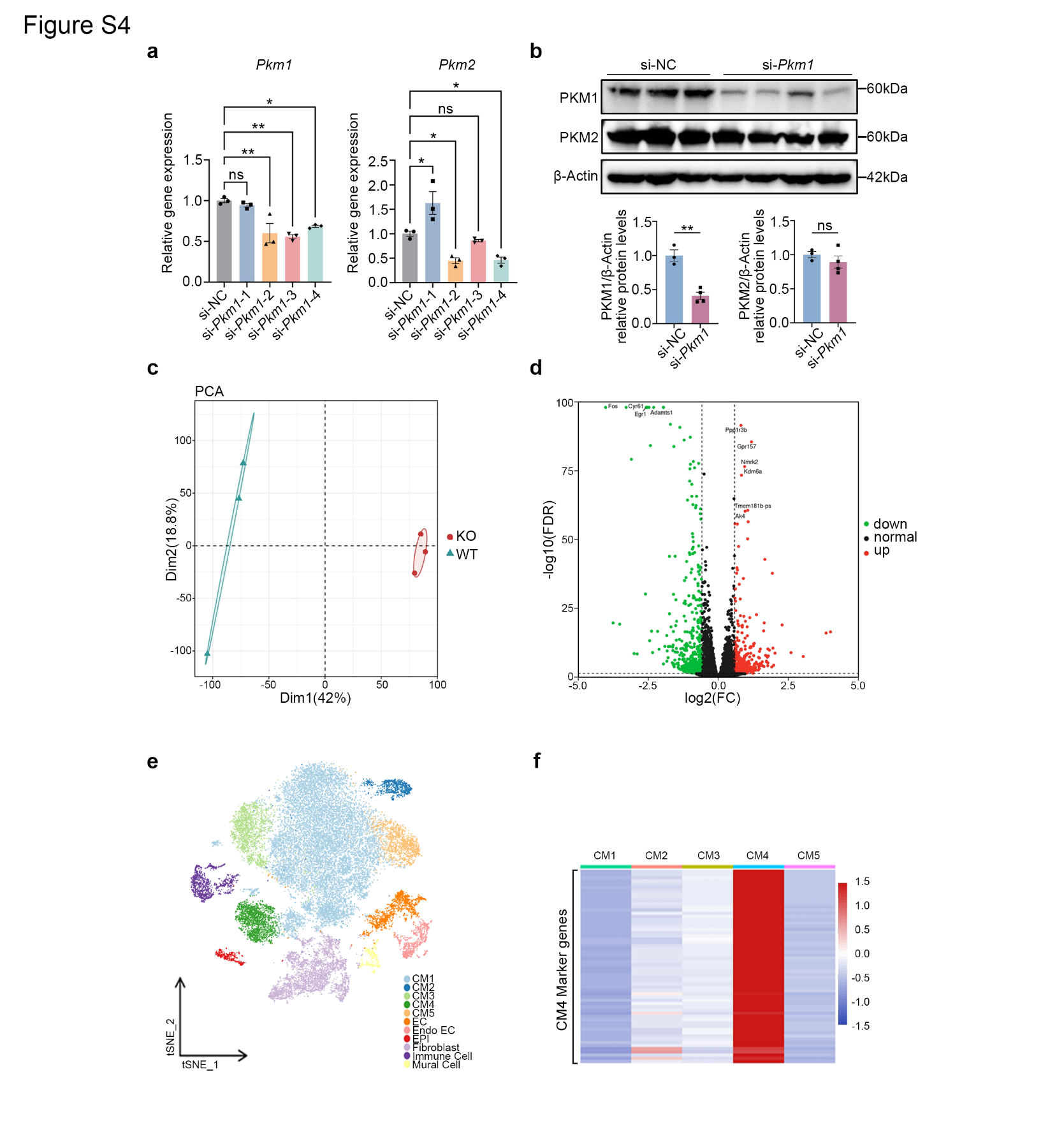


**Supplementary Fig. S4 Total RNA-seq analysis for *Pkm1*-KO hearts.**

(a) qRT-PCR analysis of *Pkm1*, *Pkm2* mRNA levels in neonatal rat ventricular cardiomyocytes (NRVCs) transfected with si-negative control (NC) or si-*Pkm1* strands for 24 h (*n* = 3 wells per group). Strand 3 was used for subsequent si-*Pkm1* experiments. (b) Western blot analysis of PKM1, PKM2 protein levels in NRVCs transfected with si-NC or si-*Pkm1* for 48 h (*n* = 3 wells/si-NC, 4 wells/si-*Pkm1*). (c) Principal component analysis (PCA) plot of transcripts for all E18.5 WT and KO heart samples. (d) Volcano plot showing differentially expressed genes (DEGs) between E18.5 WT and KO hearts. (e) Uniform manifold approximation projection (UMAP) visualization of cardiomyocyte clusters colored by identity. EC, endothelial cells; Endo EC, endocardial cells; EPI, epicardial cells. (f) Heatmap of CM4 marker genes expression in CM1-5 cells. Two-tailed unpaired Student’s t test was performed (b). One-way ANOVA, Tukey's Multiple Comparison Test was performed (a). All quantitative data are expressed as mean ± SEM, **p* < 0.05, ***p* < 0.01, ns, not significant.


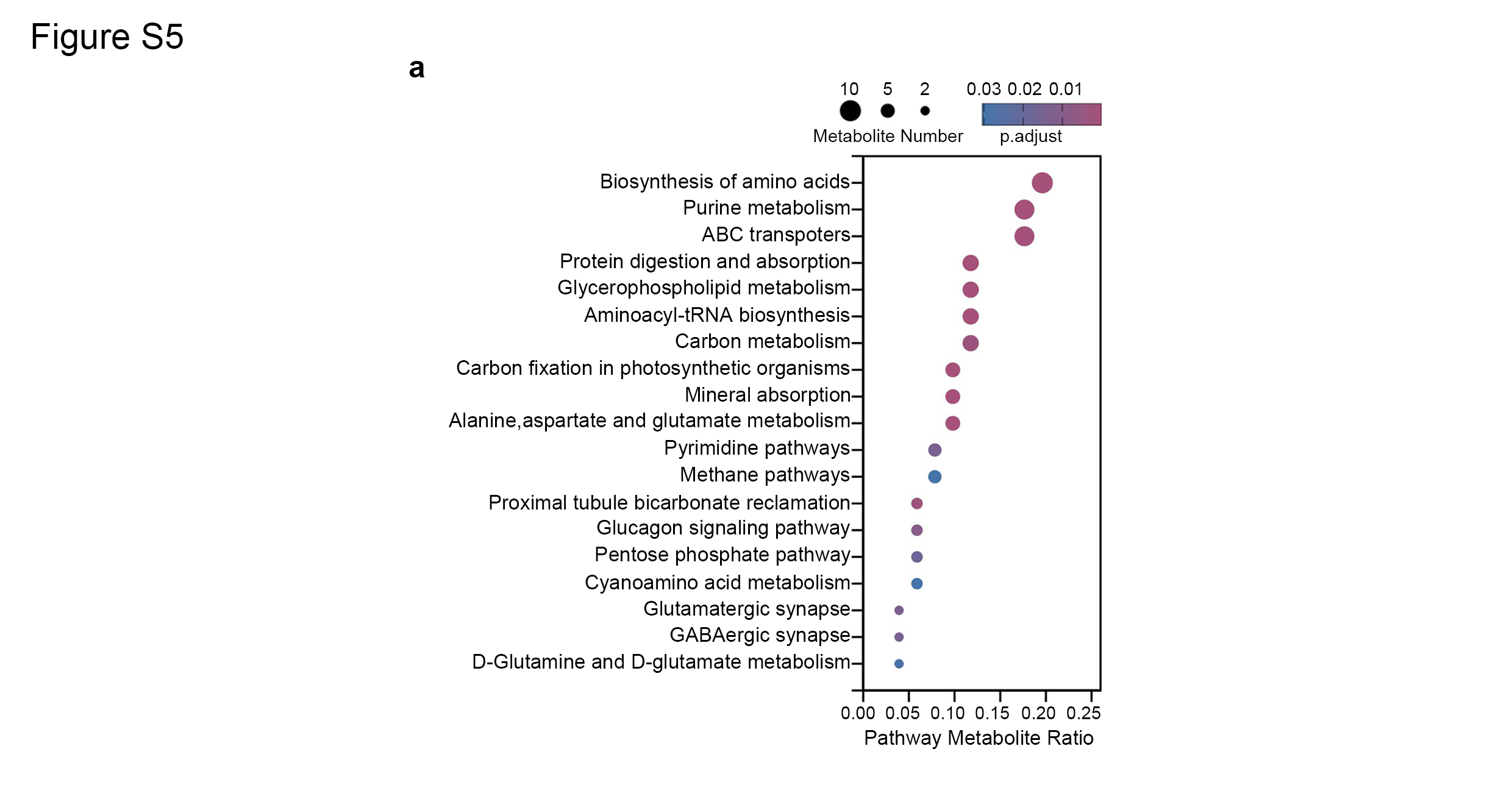


**Supplementary Fig. S5 Metabolomic analysis for *Pkm1*-KO hearts.**

(a) Metabolic pathways downregulated in *Pkm1*-KO hearts at E18.5. The dot plot represents the enriched metabolite pathways of the differential abundant metabolites.


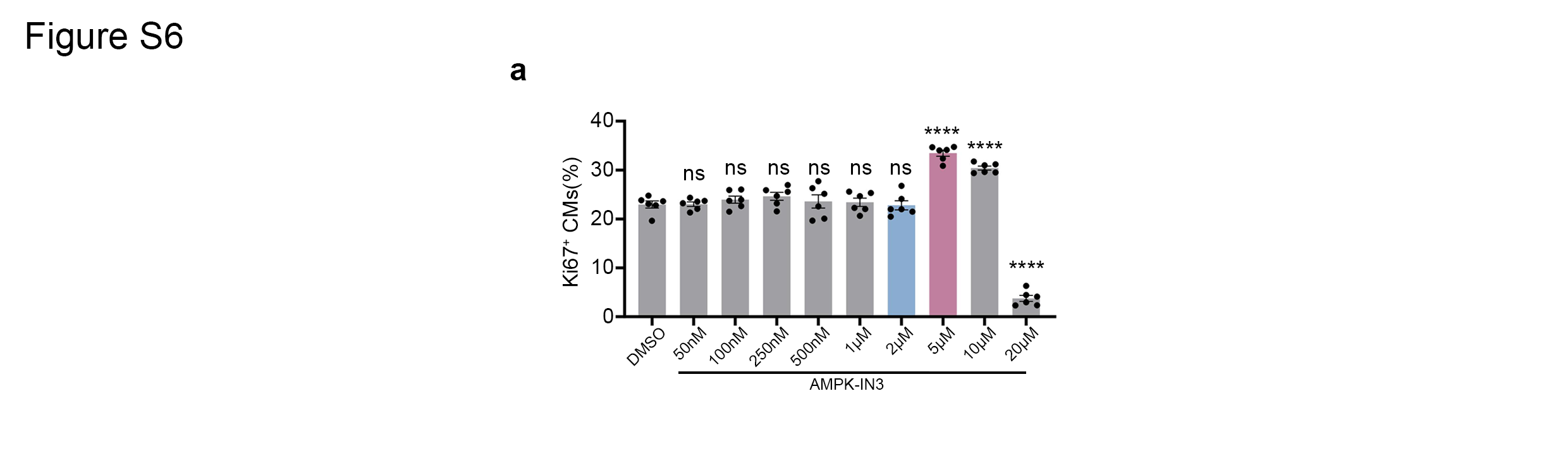


**Supplementary Fig. S6 The impact of various concentrations of AMPK-IN3 on cardiomyocyte proliferation.**

(a) Quantification of Ki67^+^ cardiomyocytes (CMs) over total CMs in NRVCs treated with dimethyl sulfoxide (DMSO) or AMPK-IN3 (from 50 nM to 20 μM) for 24 h (*n* = 6 wells per group). One-way ANOVA, Tukey's Multiple Comparison Test was performed (a). All quantitative data are expressed as mean ± SEM, **p* < 0.05, ***p* < 0.01, ****p* < 0.001, *****p* < 0.0001, ns, not significant.


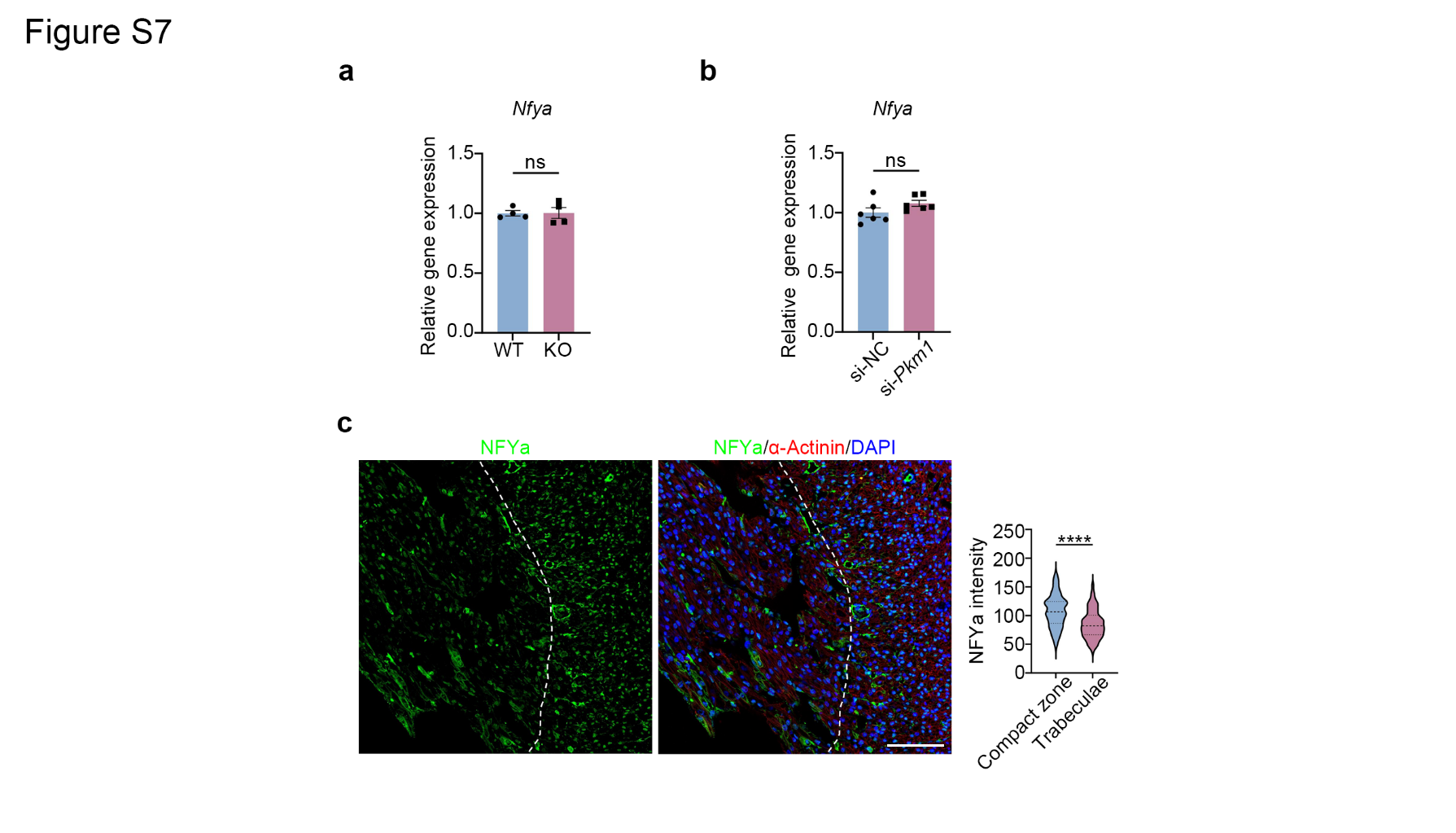


**Supplementary Fig. S7 The impact of *Pkm1* deficiency on *Nfya* RNA levels and the expression pattern of NFYa in the heart.**

(a) qRT-PCR analysis of *Nfya* mRNA levels in E18.5 WT and KO hearts (*n* = 4 hearts per group). (b) qRT-PCR analysis of *Nfya* mRNA levels in NRVCs transfected with si-NC or si-*Pkm1* for 24 h (*n* = 6 wells per group). (c) Representative images of NFYa staining and quantification of NFYa protein levels in compact zone and trabeculae zone of E18.5 WT hearts (*n* = 150 cells from 6 hearts per group). Scale bar, 100 µm. Two-tailed unpaired Student’s t test was performed (a-c). All quantitative data are expressed as mean ±SEM, **p* < 0.05, ***p* < 0.01, ****p* < 0.001, *****p* < 0.0001, ns, not significant.


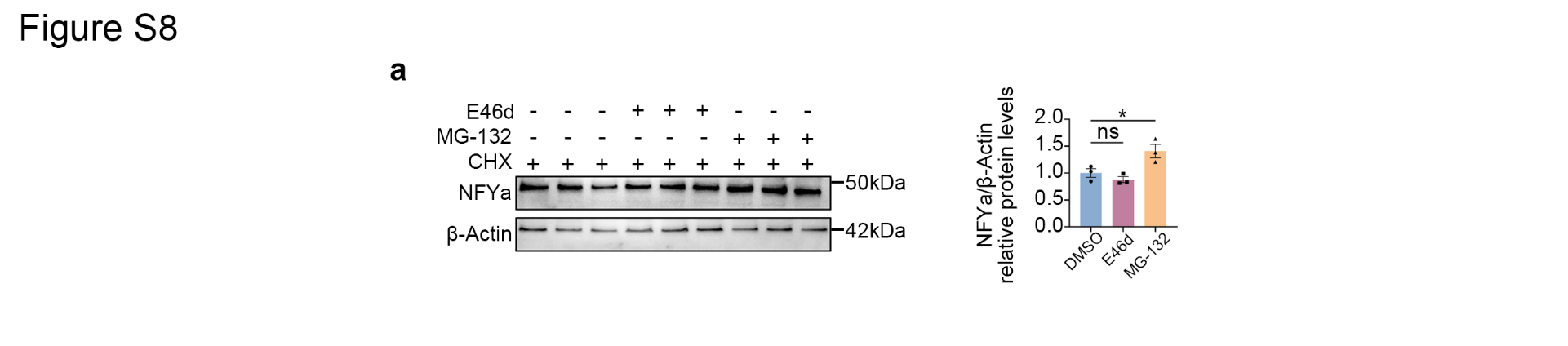


**Supplementary Fig. S8 Proteasome inhibitor MG-132 blocks NFYa degradation.**

(a) Western blot analysis and quantification of NFYa protein levels in NRVCs treated with DMSO, E64d (2.5 nM, for 6 h) or MG-132 (10 μM, for 12 h) in the presence of 1 mM cycloheximide (CHX) (*n* = 3 wells per group). One-way ANOVA, Tukey's Multiple Comparison Test was performed (a). All quantitative data are expressed as mean ± SEM, **p* < 0.05, ***p* < 0.01, ns, not significant.


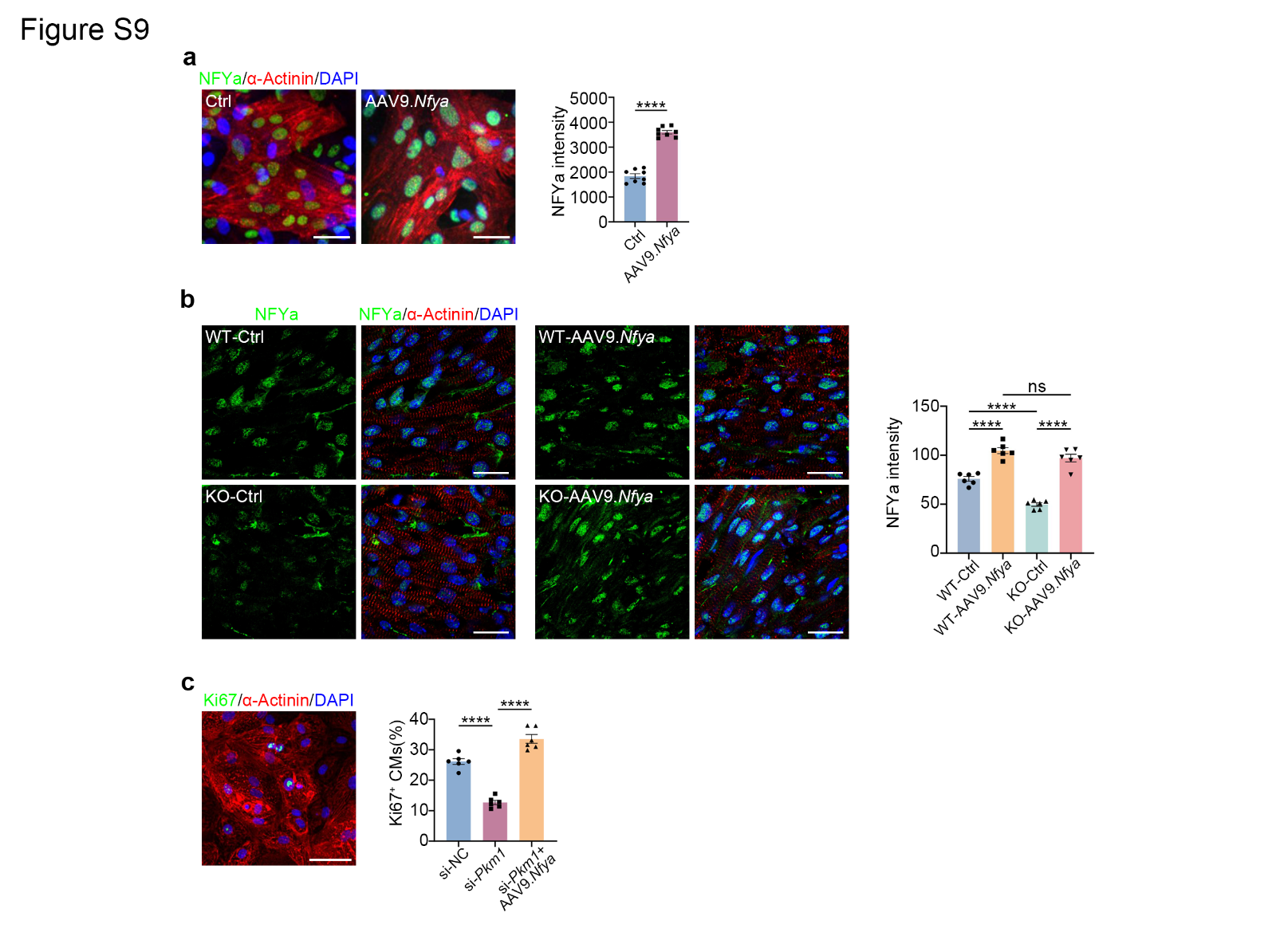


**Supplementary Fig. S9 AAV9-mediated overexpression of NFYa in vitro and in vivo.**

(a) Representative images of NFYa staining and quantification of NFYa protein levels in NRVCs with or without overexpression of NFYa for 48 h (*n* = 8 wells per group). Scale bars, 50 µm. (b) Representative images of NFYa staining and quantification of NFYa protein levels in E18.5 WT and KO hearts with or without overexpression of NFYa (*n* = 6 hearts per group). Scale bars, 20 µm. (c) Representative image of NFYa staining and quantification of NFYa protein levels in human iPSC-derived CMs transfected with si-NC or si-*Pkm1*, with or without overexpression of NFYa for 48 h (n = 6 wells per group). Scale bar, 100 µm. Two-tailed unpaired Student’s t test was performed (a). One-way ANOVA, Tukey's Multiple Comparison Test was performed (b, c). All quantitative data are expressed as mean ± SEM, **p* < 0.05, ***p* < 0.01, ****p* < 0.001, *****p* < 0.0001, ns, not significant.

**Supplementary Tables**

**Table S1. Primer and sgRNA sequences used in this study.**

| Genotyping | Forward sequence (5'-3') | Reverse sequence (5'-3') |
| --- | --- | --- |
| Pkm1-genotype | GCCTCCAGTCACTCCACAGA | TACCTGCCAGACTCCGTGAG |
| sgRNA | Sequence (5'-3') |  |
| Pkm1 | TGCTGCTAAACACTTATAAG |  |
| RT-qPCR | Forward sequence (5'-3') | Reverse sequence (5'-3') |
| Angpt2 | ATGTGGTGCAGAACCAGACA | TGGTCTGATCCAAAATCTGCT |
| Ccl2 | CCCAATGAGTAGGCTGGAGA | TCTGGACCCATTCCTTCTTG |
| Cxcl1 | CTGGGATTCACCTCAAGAACATC | CAGGGTCAAGGCAAGCCTC |
| Fas | TATCAAGGAGGCCCATTTTG | CCCCTTCTCCCAATTCTCTT |
| Mmp9 | CGTCGTGATCCCCACTTACT | AACACACAGGGTTTGCCTTC |
| Timp1 | ATCAGTGCCTGCAGCTTCTT | TCACTCTCCAGTTTGCAAGG |
| Vegf | GCGAGGCAGCTTGAGTTAAA | TCTTTCCGGTGAGAGGTCTG |
| Nfya | GGTGGACAAGGCCAAACCATC | TCTGCTGGGTTTGACCCTGC |
| Actin | AGAGGGAAATCGTGCGTGAC | CAATAGTGATGACCTGGCCGT |
| Pkm1 | GTCTGGAGAAACAGCCAAGG | TCTTCAAACAGCAGACGGTG |
| Pkm2 | GTCTGGAGAAACAGCCAAGG | CGGAGTTCCTCGAATAGCTG |
| Gapdh | AGGTCGGTGTGAACGGATTTG | TGTAGACCATGTAGTTGAGGTCA |
| Cdk1 | AGAAGGTACTTACGGTGTGGT | GAGAGATTTCCCGAATTGCAGT |
| Ccnd1 | CTCCGTATCTTACTTCAAGTGCG | CTTCTCGGCAGTCAAGGGAA |
| Ccnd2 | TGAATTACCTGGACCGTTTCTTG | AGAGTTGTCGGTGTAAATGCAC |
| Ccng1 | AAGGTCTGCGGCTTGAAACTA | CAAAGTCCCGTAATCTTGCAGT |
| Racgap1 | ACAAGACTGATGAATCACTGGAC | CCGTCGATGAACTGTCTACTGTT |
| Kif20a | CAGCGGGCTTACTCTCTGATG | TCCTCCAGTAGAGCCTGCTTG |
| Nusap1 | GAAGGCTCTCTGTACCCTGTA | GTCTTGGTCAGTGAGCACTTATG |
| Prc1 | AACTCACCTCCGGGAAATATGG | GGATATGCTTTTGAGCAGCCT |
| E2f7 | CTTGTCAACGGAGAAAACCACC | CCAGCACGTTTACGATGTCATA |
| Cdca8 | AAAAGCGAAAGGTAATCGAGGT | TGCAGATCGAAGATTCTTATGGC |
| Tpx2 | CACACCGTTGAAGGCAGTTG | ACAGCTCTCTTAGCATCCAGG |
| Top2a | AACAAAGGGACCCAAAAATGTCT | TGTGTTCAACAACAGGGATTCC |

**Table S2. Mutation analysis of predicted off-target loci in *Pkm1*-KO mice.**

| Rank | Candidate off-target sequence | CFD score | Chromosome | Gene context | Indel frequency |
| --- | --- | --- | --- | --- | --- |
| 1 | AACTGATAAACACTTATAAAAGG | 0.736607143 | chr6 | Intergenic | 1% |
| 2 | GGCTAATAGACACTTATAAGTGG | 0.513181505 | chrX | Intergenic | 0.60% |
| 3 | GGCTAATATACACTTATAAGTGG | 0.461863354 | chr12 | Intergenic | 0.90% |
| 4 | TGCTAATATTCACTTATAAGTGG | 0.42605042 | chr12 | Intergenic | 1.10% |
| 5 | GGCTGCAAAACATTTACAAGTGG | 0.413554987 | chr2 | Exon of *Sema6d* | 0.90% |
| 6 | TGCTGATATACACATATAAAGGG | 0.323341837 | chr12 | Intergenic | 0.70% |
| 7 | TGCTGTTAGGCACTTATAAAAGG | 0.322420635 | chr12 | Intron of *Rgs6* | 0.90% |
| 8 | TACTGTTAAAAACTTAAAAGGGG | 0.314285714 | chr16 | Intron of *Hacd2* | 1.40% |
| 9 | TGCAGATATACACTTATAAGTGG | 0.265306122 | chr17 | Intergenic | 0.90% |
| 10 | TTCAGCAAAACACTTATAAGGGG | 0.265151515 | chr2 | Intron of *Arhgap15* | 1.40% |
| 11 | TGCTACTACTCACTAATAAGTGG | 0.252985404 | chrX | Intergenic | 0.70% |
| 12 | CCCAACTAAACACTTATAAGTGG | 0.244897959 | chr11 | Intron of *Gpatch8* | 1.40% |
| 13 | GGCAGCGAATCACTTATAAGTGG | 0.2364117 | chr1 | Intergenic | 0.70% |
| 14 | AGCTACTAAACACTTAAAACAGG | 0.198095238 | chr15 | Intergenic | 1.20% |
| 15 | TACTGGAAAACACTTAAAAGTGG | 0.197435897 | chr7 | Intergenic | 1.60% |

**Table S3. Antibodies and compounds used in this study.**

|  | Company | Catalog number |
| --- | --- | --- |
| PKM1 | CST | 7067S |
| PKM2 | CST | 4053S |
| α-Tubulin | Proteintech | 66031-1-Ig |
| α-Actinin | Sigma | A7811 |
| Ki67 | CST | 9129T |
| pH3 | CST | 9706L |
| AURKB | CST | 3094S |
| p-AMPK | CST | 2535T |
| AMPK | Proteintech | 10929-2-AP |
| NFYa | Santa Cruz | sc-17753 |
| α-Actinin | Thermo Fisher Scientific | 701914 |
| GAPDH | CST | 5174S |
| β-Actin | CST | 4970T |
| H3 | Abcam | ab1791 |
| AMPK-IN1 | MCE | HY-120904 |
| AMPK-IN3 | MCE | HY-151361 |
| MG132 | Selleck | S2619 |
| CHX | Selleck | S7418 |
| E64d | Selleck | S7393 |
