# Supplementary figures and images for: PKM1 is required for embryonic cardiomyocyte proliferation through energetic regulation of NFYa stability
Source: Natl Sci Rev. 2025 Sep 25;13(1):nwaf408. doi: 10.1093/nsr/nwaf408 (PMC12796809; doi:10.1093/nsr/nwaf408)

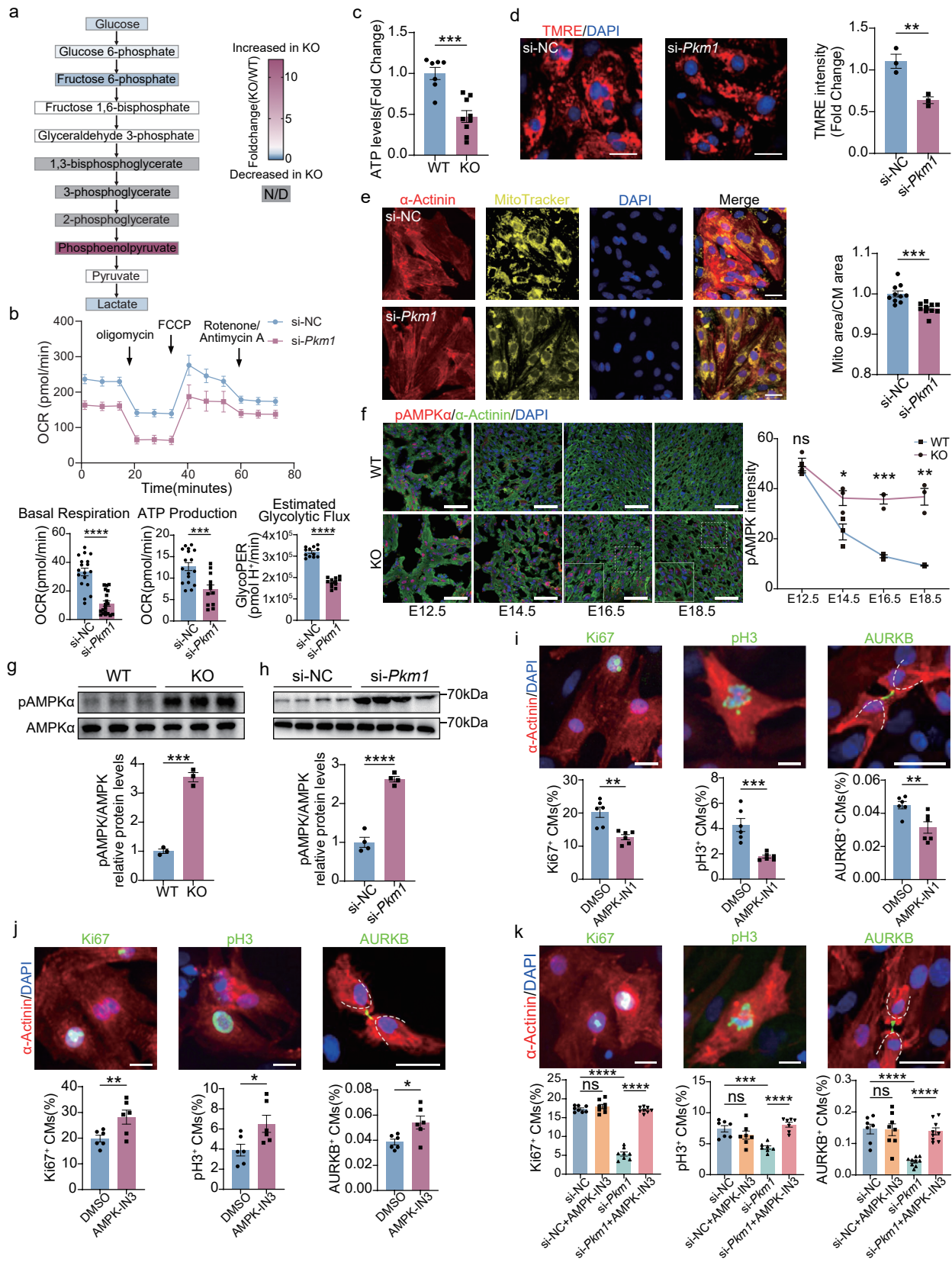

Supplement: nwaf408_Supplemental_Files [file nwaf408_supplemental_files.zip › Figure3.170mm.author.pdf]

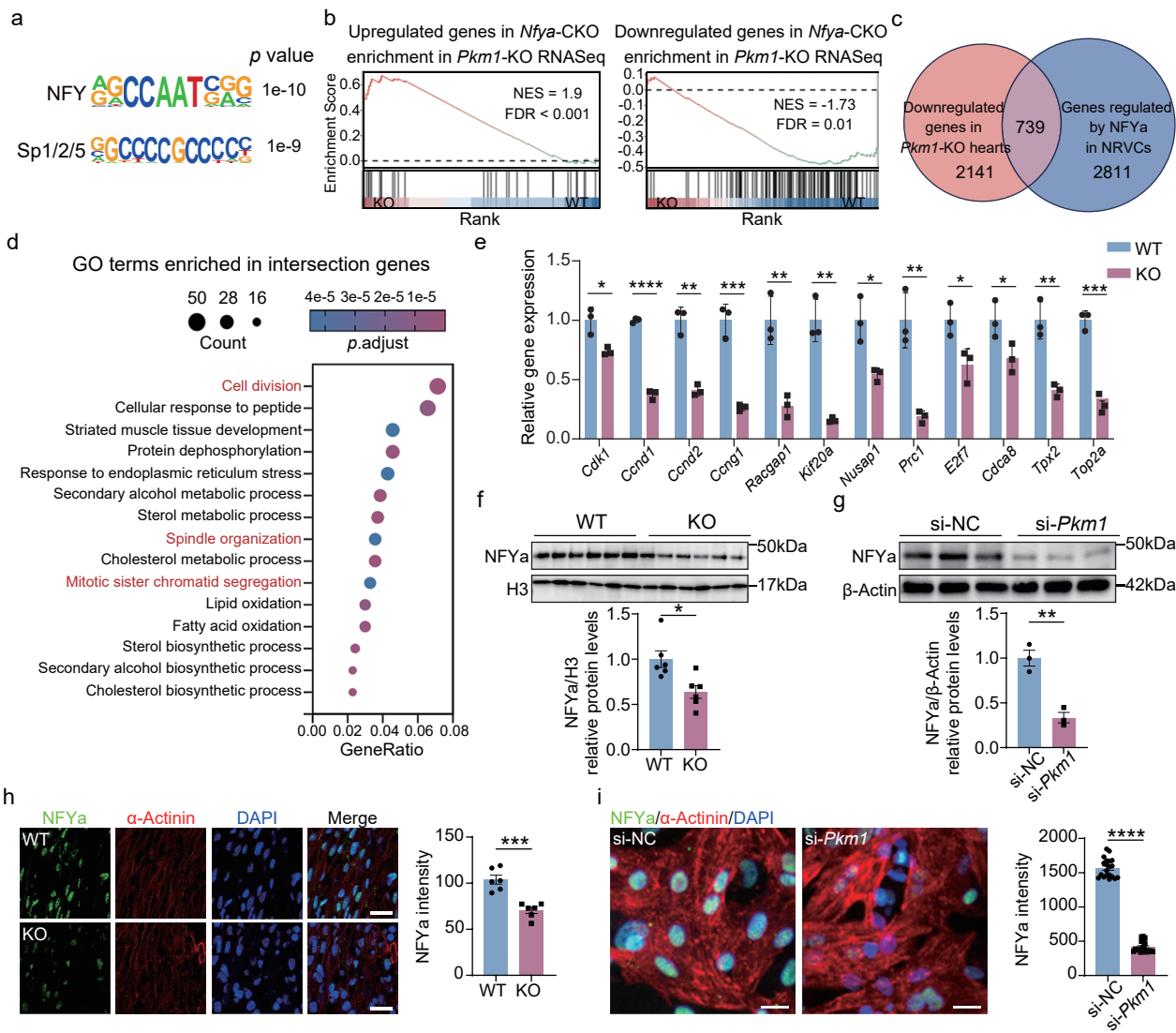

Supplement: nwaf408_Supplemental_Files [file nwaf408_supplemental_files.zip › Figure4.170mm.author.0922.pdf]

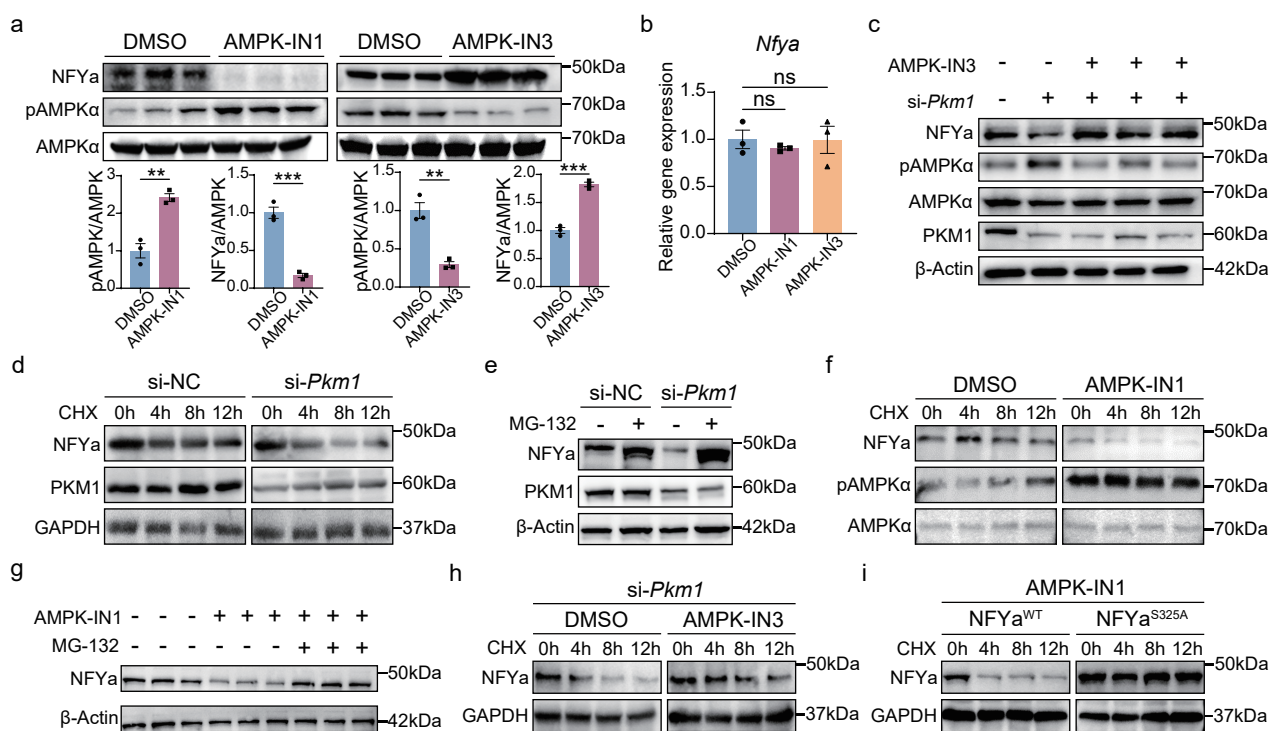

Supplement: nwaf408_Supplemental_Files [file nwaf408_supplemental_files.zip › Figure5.170mm.author.pdf]
